# Progressive Cactus is a multiple-genome aligner for the thousand-genome era

**DOI:** 10.1038/s41586-020-2871-y

**Published:** 2020-11-11

**Authors:** Joel Armstrong, Glenn Hickey, Mark Diekhans, Ian T. Fiddes, Adam M. Novak, Alden Deran, Qi Fang, Duo Xie, Shaohong Feng, Josefin Stiller, Diane Genereux, Jeremy Johnson, Voichita Dana Marinescu, Jessica Alföldi, Robert S. Harris, Kerstin Lindblad-Toh, David Haussler, Elinor Karlsson, Erich D. Jarvis, Guojie Zhang, Benedict Paten

**Affiliations:** 1 UC Santa Cruz Genomics Institute, UC Santa Cruz, Santa Cruz, CA USA; 2 BGI-Shenzhen, Beishan Industrial Zone, Shenzhen, China; 3 Section for Ecology and Evolution, Department of Biology, University of Copenhagen, Copenhagen, Denmark; 4 BGI Education Center, University of Chinese Academy of Sciences, Shenzhen, China; 5 State Key Laboratory of Genetic Resources and Evolution, Kunming Institute of Zoology, Chinese Academy of Sciences, Kunming, China; 6 Broad Institute of Harvard and Massachusetts Institute of Technology (MIT), Cambridge, MA USA; 7 Science for Life Laboratory, Department of Medical Biochemistry and Microbiology, Uppsala University, Uppsala, Sweden; 8 Department of Biology, The Pennsylvania State University, University Park, PA USA; 9 Howard Hughes Medical Institute, Chevy Chase, MD USA; 10 Program in Molecular Medicine, University of Massachusetts Medical School, Worcester, MA USA; 11 Bioinformatics and Integrative Biology, University of Massachusetts Medical School, Worcester, MA USA; 12 Laboratory of Neurogenetics of Language, The Rockefeller University, New York, NY USA; 13 Center for Excellence in Animal Evolution and Genetics, Chinese Academy of Sciences, Kunming, China; 14 China National GeneBank, BGI-Shenzhen, Shenzhen, China

**Keywords:** Genome informatics, Phylogeny, Software, Comparative genomics

## Abstract

New genome assemblies have been arriving at a rapidly increasing pace, thanks to decreases in sequencing costs and improvements in third-generation sequencing technologies^1–3^. For example, the number of vertebrate genome assemblies currently in the NCBI (National Center for Biotechnology Information) database^4^ increased by more than 50% to 1,485 assemblies in the year from July 2018 to July 2019. In addition to this influx of assemblies from different species, new human de novo assemblies^5^ are being produced, which enable the analysis of not only small polymorphisms, but also complex, large-scale structural differences between human individuals and haplotypes. This coming era and its unprecedented amount of data offer the opportunity to uncover many insights into genome evolution but also present challenges in how to adapt current analysis methods to meet the increased scale. Cactus^6^, a reference-free multiple genome alignment program, has been shown to be highly accurate, but the existing implementation scales poorly with increasing numbers of genomes, and struggles in regions of highly duplicated sequences. Here we describe progressive extensions to Cactus to create Progressive Cactus, which enables the reference-free alignment of tens to thousands of large vertebrate genomes while maintaining high alignment quality. We describe results from an alignment of more than 600 amniote genomes, which is to our knowledge the largest multiple vertebrate genome alignment created so far.

## Abstract (web summary)

The Progressive Cactus program can create reference-free alignments of hundreds of large vertebrate genomes efficiently, and is used for the alignment of more than 600 amniote genomes.

## Main

Comparative genomics analyses, including species-tree inference^7,8^, comparative annotation^9,10^, and selection detection^11,12^, require genome alignments. Multi-species genome alignment involves creating a mapping from each region of each genome to a corresponding region in each other genome, taking into account the possibility of complex rearrangements and copy number changes^13^. Genome aligners are one of the most fundamental tools used in comparative genomics, but because the problem is difficult, different aligners frequently give different results^14^, and many intentionally limit the alignments they produce to simplify the problem. Two of the most common limitations are ‘reference bias’, the result of constraining a multiple alignment to only regions present in a single reference genome, and restricting the alignment to be ‘single-copy’, which allows only a single alignment in any column in any given genome, causing the alignment to miss multiple-orthology relationships created by lineage-specific duplications. Cactus^6^ is a genome alignment program that has neither of these restrictions; it can generate a reference-free multiple alignment that allows the detection of multiple-orthology relationships.

The version of Cactus available in 2012 performed very well in the Alignathon^14^, an evaluation of genome aligners. However, the runtime of that initial iteration of Cactus scaled quadratically with the total number of bases in the alignment problem, making alignment of more than about ten vertebrate genomes completely impractical. To address these difficulties, we present fundamental changes to the Cactus process that incorporate a progressive alignment strategy^15^, which changes the runtime of the alignment to scale linearly with the number of genomes. We show that the result, which we call Progressive Cactus, is an aligner that retains state-of-the-art accuracy, and continues to lack reference bias, but which is tractable to use on hundreds to thousands of large, vertebrate-sized input genomes. Progressive Cactus has been developed over several years, and has already been successfully used as an integral component of high-profile comparative genomics projects^16–20^.

## Progressive Cactus

The new Progressive Cactus pipeline is freely available and open source. The only inputs needed are a guide tree and a FASTA file for each genome assembly.

The key innovation of Progressive Cactus is to adapt the classic ‘progressive’ strategy (used in collinear multiple alignment for decades) to a whole-genome alignment setting. Progressive aligners use a ‘guide tree’ to recursively break a multiple alignment problem into many smaller sub-alignments, each of which is solved independently; the resulting sub-alignments are themselves aligned together according to the tree structure to create the final alignment. Progressive alignment has been successfully applied to whole-genome alignment before—for example, by progressiveMauve^21^ and TBA/MULTIZ^22^. Cactus now follows a similar strategy, with the key innovation being that Progressive Cactus implements a progressive-alignment strategy for whole-genome alignment using reconstructed ancestral assemblies as the method for combining sub-alignments. This strategy (analogous to the MAVID^23^ strategy of using ancestral reconstruction in collinear multiple alignment) not only results in a much faster alignment runtime but also produces ancestral reconstructions.

**Fig. 1:**
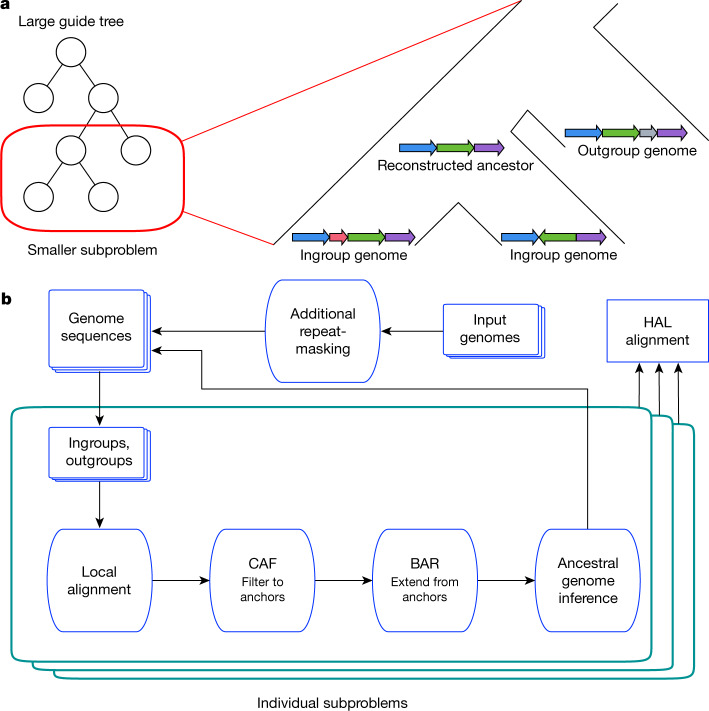
The alignment process within Progressive Cactus. **a**, A large alignment problem is split into many smaller subproblems using an input guide tree. Each subproblem compares a set of ingroup genomes (the children of the internal node to be reconstructed) against each other as well as a sample of outgroup genomes (non-descendants of the internal node in question). **b**, Flowchart represents the phases in which the overall alignment, as well as each subproblem alignment, proceeds through. The end result is a new genome assembly that represents the Progressive Cactus reconstruction of the ancestral genome, and an alignment between this ancestral genome and its children. After all subproblems have been completed, the parent–child alignments are combined to create the full reference-free alignment in the HAL^27^ format.

Figure 1a shows the overall organization of the Progressive Cactus process. The guide tree, which need not be fully resolved (binary), is used to recursively split a large alignment problem (comparing every genome to every other genome) into many small subproblems, each of which compares only a small number (usually 2–5) of genomes against one another. The purpose of each subproblem is to reconstruct an ancestral assembly at each internal node in the guide tree, as well as to generate alignments between that internal node’s children and its ancestral reconstruction. The ancestral assemblies are then used as input genomes in subproblems further up the tree, and the parent–child alignments are later combined to produce the full alignment. Two sets of genomes are considered: the children of the internal node (which we call the ‘ingroup genomes’), and a set of non-descendants of that node (the ‘outgroup genomes’). The ingroup genomes form the core alignment relationship being established at this node. The outgroup genomes serve to answer the question of what sequence from the ingroups is also present in the ancestor (whether an indel among the ingroups is likely to be a deletion rather than an insertion), and in how many copies (whether a duplication predates or postdates the speciation event the node represents). The outgroups also provide information for guiding the ancestral assembly by providing order-and-orientation information, as well as base-level information when generating ancestral sequences. These genome sets are used as the input to the main subproblem workflow (Fig. 1b).

**Extended Data Fig. 1:**
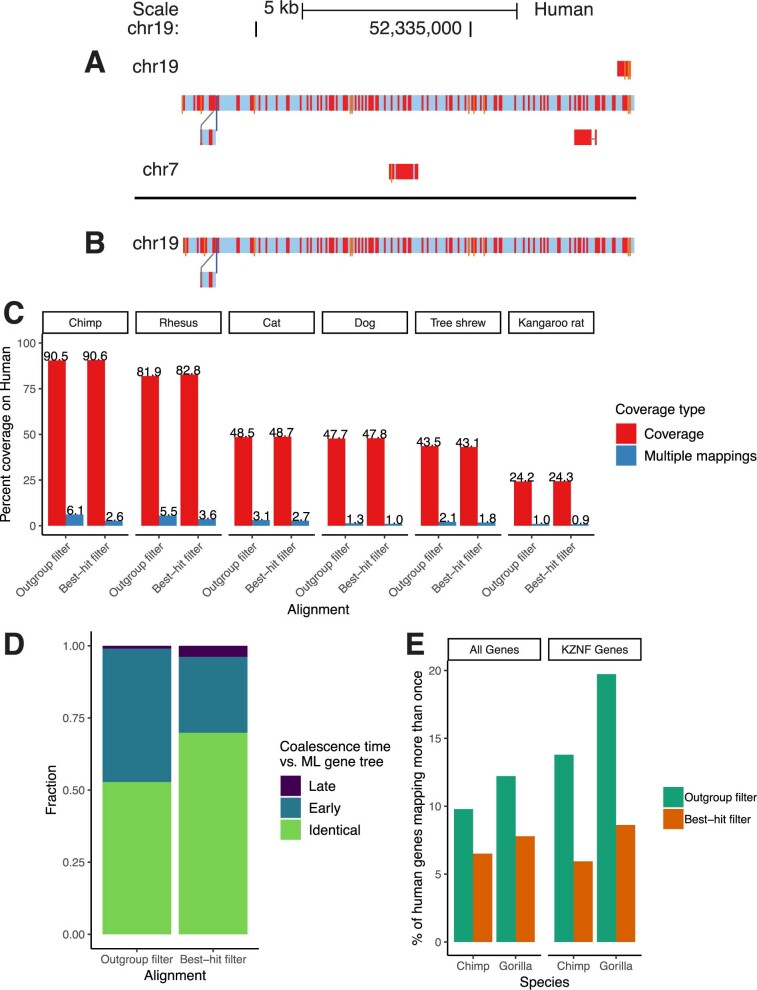
Results from improved paralogue filtering. **a**, **b**, A sample snake track^64^ within a recently duplicated region before (**a**) and after (**b**) the filtering change. Nucleotide substitutions are shown as red bars, and insertions are shown as thin orange bars. **c**, Coverage results from two alignments of identical assemblies using the outgroup and best-hit filtering methods. Multiple-mappings: sites that map to two or more sites on the target genome. **d**, Results from comparing phylogenetic trees implicit in the HAL alignment to ML re-estimated trees of the same regions. ‘Early’ coalescences indicate that too many duplication events have been created in the reconciled trees, and ‘late’ indicates that too many loss events have been created. **e**, Percentage of human genes that map more than once to the chimp/gorilla genomes in two CAT^9^ annotations using alignments created with the outgroup and best-hit filtering methods. KZNF, KRAB zinc-finger genes.

Each individual subproblem follows a similar procedure to the original Cactus process. The subproblem procedure begins with a set of pairwise local alignments generated via the sensitive pairwise local-alignment program LASTZ^24^. These pairwise alignments are then filtered and combined into a cactus graph representing an initial multiple alignment using the previously described CAF algorithm^6^—although we note important changes to the filtering in Methods and Extended Data Fig. 1—to attempt to recover the homologies that date to the most recent common ancestor of the ingroup genomes. The initial alignment is refined using the previously described BAR algorithm^6^ to create a more complete alignment. The ancestral assembly is then created by ordering the blocks in this final alignment and establishing a most-likely base call for each column in each block. The resulting ancestral sequence is then fed into later subproblems (unless the root of the guide tree has been reached, which ends the alignment process).

As a practical matter, Progressive Cactus uses the Toil^25^ workflow framework to organize and distribute its computational tasks. Although genome alignment is a computationally intensive task, using Toil, we can break up the problem into small pieces that can work in heterogeneous compute environments, playing to the advantages of both cheap CPU-rich machines and more expensive memory-rich machines. Because it runs on Toil and supports container execution via Docker and Singularity^26^, Progressive Cactus can be run on many different environments: single machines (for small alignments), conventional clusters, and commercial clouds.

**Extended Data Fig. 2:**
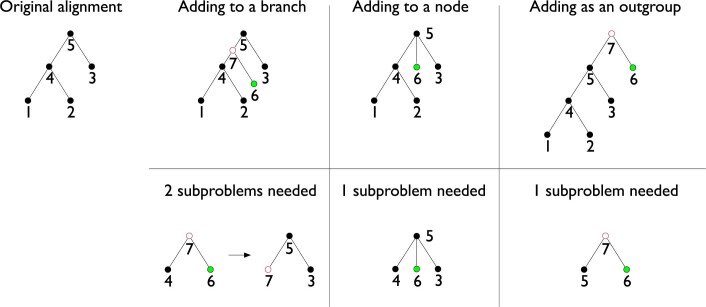
Methods of adding a genome to a Progressive Cactus alignment. The top row shows the different ways of adding a new genome given its phylogenetic position, and the bottom row shows what subproblems would need to be computed for the new genome to be properly merged into the existing alignment. Green circles represent a new genome, and red circles represent newly reconstructed genomes.

Given the rate of arrival of new assembly versions and newly sequenced genomes, adding new information to an alignment without recomputing it from scratch is valuable, especially for large alignments in which recomputing the entire alignment is often cost-prohibitive. Progressive Cactus, combined with special functionality in the HAL toolkit^27^, therefore supports the addition and removal of genomes from the alignment by taking advantage of the tree structure of the progressive alignments it produces (Methods, Extended Data Fig. 2, Extended Data Table 1).

## Evaluation on simulated data

The Alignathon simulated datasets^14^ have been aligned with many competing genome aligners and have a known truth set, providing a way to compare Progressive Cactus against other genome aligners. Progressive Cactus produces alignments with higher accuracy for both simulated primate (*F*_1_ score of 0.989) and mammal (*F*_1_ score of 0.795) clades than any aligner that participated in the Alignathon (Supplementary Tables 1, 2), including the original version of Cactus.

**Fig. 2:**
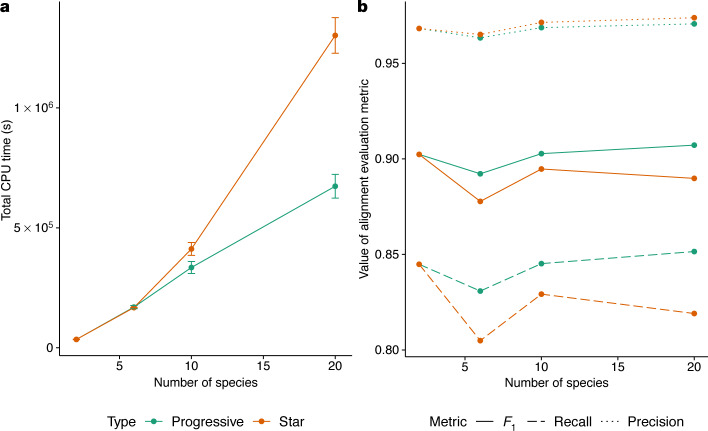
Comparing alignments of varying numbers of simulated genomes using Progressive Cactus. **a**, The progressive mode of Progressive Cactus is shown, versus the mode without progressive decomposition that is similar to that previously described^6^ (‘star’). The average total runtime of the two alignment methods across three runs is shown. Data are mean and s.d. The runtime is identical when aligning two genomes as the alignment problem is not further decomposed, but the linear scaling of the progressive mode means it is much faster with large numbers of genomes than the quadratic scaling required without progressive alignment. **b**, The precision, recall and *F*_1_ score (harmonic mean of precision and recall) of aligned pairs for each alignment compared with pairs from the true alignment produced by the simulation. Source data

To evaluate the improvements in quality and runtime of the alignments produced using the new progressive alignment strategy, we simulated the evolution of twenty 30-megabase genomes using Evolver (https://www.drive5.com/evolver) along a tree of catarrhines. We ran two alignment strategies—one using a fully resolved binary guide tree (which takes full advantage of the new progressive mode), and one using a fully unresolved star guide tree (which is similar to the originally published version of Cactus)—across variously sized subsets of genomes roughly evenly spaced throughout the catarrhine tree. The alignments using the progressive strategy finished more quickly, with the speed improvement growing larger with the increasing number of species (for example, a 15% reduction in runtime for 10 species and 48% for 20 species), owing to its linear runtime scaling, as opposed to the quadratic scaling of the star-tree (Fig. 2a). The progressive strategy is also more accurate than the star strategy (Fig. 2b) and maintains accuracy as the number of species (and therefore nodes in the tree) increases.

## Effect of the guide tree

Because Progressive Cactus uses an input guide tree to decompose the alignment problem, the guide tree can potentially impact the resulting alignment. This could be problematic when the exact species tree relating the input set of genomes is unknown or controversial. However, we reduce any effect of the guide tree by including a great deal of outgroup information, including multiple outgroups when possible. To quantify the effect of the guide tree on a large alignment with an uncertain species tree, we created four alignments of a set of 48 avian species (Supplementary Table 3), which we subset down to a single chromosome (chromosome 1). The avian species tree is still being actively debated^28,29^ and there are different plausible hypotheses, making birds an excellent test case with no single clearly correct guide tree. We aligned these birds using four different guide trees: two trees that represent two different hypotheses about the avian species tree^28,29^, one consensus tree between the former two trees, and one tree that was randomly permuted from one of the previously published trees^29^ (Methods, Supplementary Fig. 1). The four alignments were highly similar, with an average of 98.5% of aligned pairs identical between any two different alignments (Extended Data Table 2).

We further examined whether these small differences in the guide tree affect some species more than others. For any pair of these 48 species, the *F*_1_ score for aligned pairs between the previously published^28,29^ alignments was at least 0.955 (Supplementary Fig. 2). As an example, the phylogenetic relationship between the species *Cuculus canorus*, *Chlamydotis macqueenii* and *Tauraco erythrolophus* is different in the guide tree based on Prum et al.^28^ than that based on Jarvis et al.^29^ (Supplementary Fig. 3). The *F*_1_ score for aligned pairs within this clade between the two alignments was 0.972, lower but comparable to the score for a similar clade that had an identical phylogenetic relationship in both trees, 0.982 (for *Merops nubicus*, *Picoides pubescens* and *Buceros rhinoceros*).

## Effect of assembly quality on alignment

Our progressive approach means that the alignment between two genomes distant in the guide tree is informed by the reconstructions of the ancestral genomes along the path, which is in turn formed using data from other genomes in the tree. To evaluate the practical effect of differing quality of input assemblies, we created two alignments of 11 boreoeutherian mammal species, 7 of which represented either high-quality assemblies in one alignment (using modern assemblers and often long-read data) or lower quality assemblies in the other alignment (usually using much older shorter-read technologies) (Supplementary Table 4). The remaining four assemblies were held constant to facilitate a comparison between the two alignments. Despite alignment differences between the long-read and short-read assemblies (Supplementary Table 5), the alignment between these four assemblies was similar in both datasets (for example, 0.855 Jaccard similarity between induced pairwise human–dog alignments) (Supplementary Fig. 4), a level of similarity higher than seen between alignment strategies, indicating that the progressive alignment strategy can tolerate poor assemblies. Reinforcing this, comparing the induced pairwise alignments of human–dog to direct pairwise alignments computed using the established chains and nets pipeline^30^, we find the same level of Jaccard similarity for both the high- and low-quality assembly alignments (Supplementary Fig. 5). Of the aligned pairs in the induced pairwise Progressive Cactus alignments, 82% were found in the chains and nets alignment, and, vice versa, 78% of pairs in the chains and nets alignment were found in each Progressive Cactus alignment. Concordant results were found comparing human–mouse pairwise alignments (Supplementary Fig. 5).

## 600-way amniote alignment

**Fig. 3:**
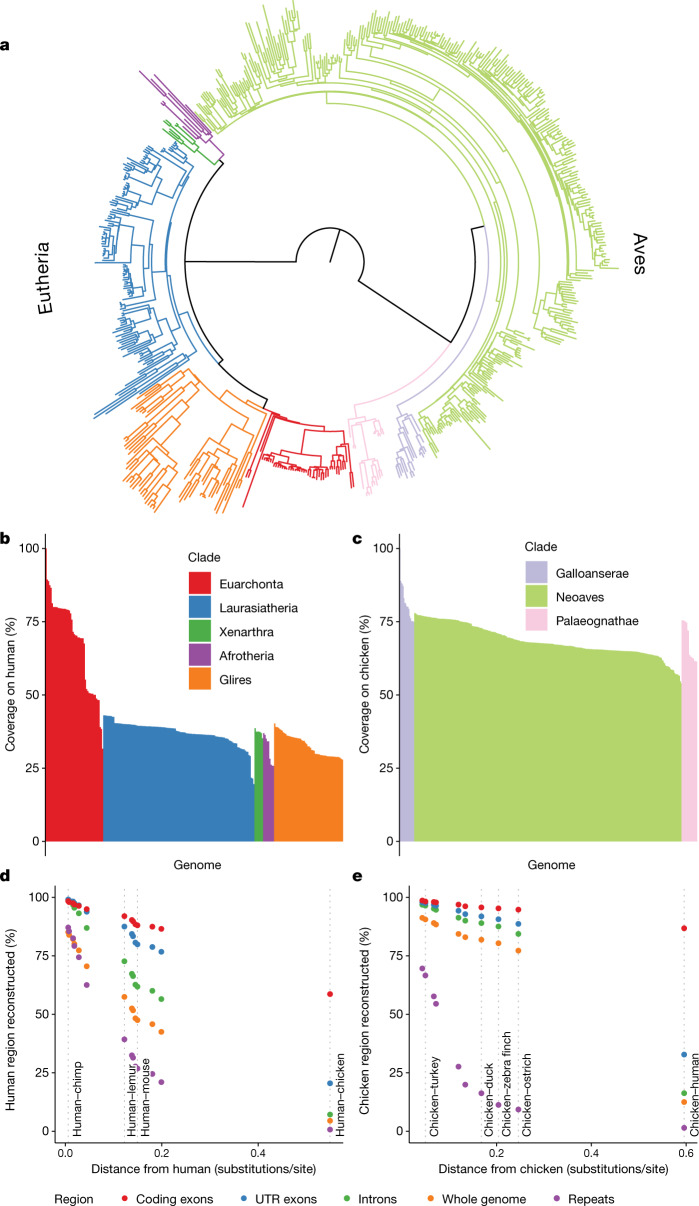
Analysing the 600-way amniote alignment. **a**, The species tree relating the 600 genomes. Branches are coloured by clades as in **b** and **c**. **b**, Percentage coverage on human within the eutherian mammals, grouped by clade from highest to lowest coverage. **c**, As in **b**, but for coverage on chicken within the avian alignment. **d**, Percentage of various regions within the human genome mappable to each ancestral genome reconstructed along the path leading from human to the root. The positions of selected ancestors are labelled by dotted lines to indicate useful taxonomic reference points as context. UTR, untranslated region. **e**, As in **d**, but for the path of reconstructed ancestors between chicken and the root. Source data

To demonstrate Progressive Cactus, we present results from an alignment of 605 amniote genomes, relating in a reference-free manner to more than 1 trillion bases of DNA across hundreds of millions of years of genome evolution (an estimated 35.4 neutral substitutions per site). The amniote-wide alignment combines two smaller alignments: one created for the initial release of the Zoonomia project^31^, which includes 242 placental mammals representing most eutherian mammal families, and one for the Bird 10,000 genomes (B10K) project^32^, which includes 363 avians, also representing most bird families. The overall topology is shown in Fig. 3a. To our knowledge, this represents the largest whole-genome alignment created so far. Table 1 contains aggregate statistics on this alignment.

**Table 1:** Aggregate statistics for the 600-way alignment

Alignment	No. of genomes	Total bases	Instance-hours	Core-hours	Common ancestor size
Zoonomia	242	669 billion	68,166	1.9 million	1.73 Gb
B10K	363	400 billion	5,302	0.2 million	1.13 Gb
Combined	605	1.07 trillion	73,692	2.1 million	181 Mb

The increase in computational work for the mammal alignment compared with the bird alignment is largely caused by the increase in the pairwise alignment phase runtime because it scales quadratically with the size of the genomes being aligned.

Coverage within the 600-way alignment closely tracks phylogenetic distance and genome size; for example, a median coverage on human of 2.3 gigabases (Gb) from Euarchonta mammalian species, versus 1.2 Gb and 1.0 Gb from more distant Laurasiatheria and Glires mammalian species, respectively (Fig. 3b, c). The ancestral reconstructions within the 600-way alignment are highly complete, especially for functional sequence: 86% of human coding bases are represented in our reconstruction of the ancestor of all placental mammals, whereas 95% of chicken coding bases are represented in our reconstruction of the common ancestor of avians (Fig. 3d, e). Owing to the long branch length (approximately 0.7 substitutions-per-site divergence between the two clades), the amniote (human–chicken) ancestral assembly has a much lower proportion of reconstructed sequence than its immediate children, the avian and eutherian mammal ancestors, for example, retaining 16.3% of chicken intron bases versus 84.4% in the avian ancestor, and 7.2% of human intron bases versus 56.5% in the eutherian ancestor. However, coding bases are still well retained (86.8% from chicken and 58.7% from human). The ancestral assemblies consistently contain a relatively higher proportion of sequence for avians than for mammals even across similar phylogenetic distances, consistent with a more conservative mode of genome evolution in avians that is influenced by lower repeat counts and denser gene content^33^.

**Extended Data Fig. 3:**
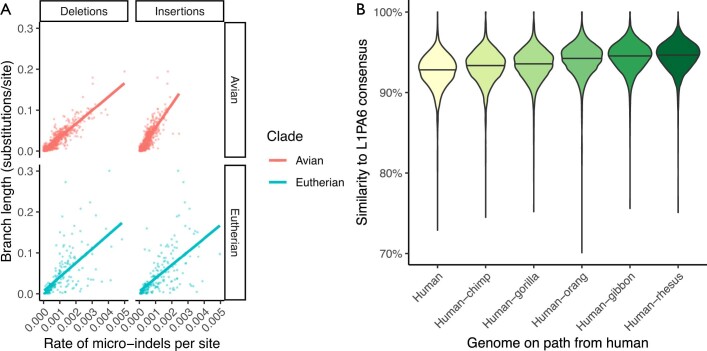
Analysing insertions, deletions and L1PA6 repeats in the 600-way alignment. **a**, Rates of micro-insertions and -deletions (micro-indels) along each branch within the 600-way alignment, compared to conventional substitutions/site branch length. The data from avian and eutherian branches are separated. Lines show a best-fit linear model for each category. **b**, Violin plot showing the increasing similarity to consensus of L1PA6 elements within reconstructed ancestral genomes along the path to the emergence of modern L1PA6 elements (in the human-rhesus ancestor). Horizontal lines indicate the median values.

The ancestral reconstructions provide a history of substitution, indel and rearrangement events. Although this history is by its nature only a hypothetical reconstruction of the true history of genome evolution along the tree, it is accurate enough to be useful. To demonstrate the utility of the indel history, we examined rates of small (less than or equal to 20 base pairs (bp)) insertion and deletion events in the 600-way alignment. As expected from previous studies^16,34^, the rate of small indels in any given branch was correlated with the rate of nucleotide substitution (an *R*^2^ value of 0.69 for insertions and 0.80 for deletions in avians, and 0.39 and 0.40, respectively, for eutherians), although the relative rates remained lower for insertions (1.2% of the substitution rate for both clades) and for deletions (2.4% and 1.2% of the substitution rate for avians and eutherians, respectively). Notably, we observe similar rates of deletions between eutherian and avian lineages, but evidence of a slightly increased rate of insertions in avians (Extended Data Fig. 3a). The ancestral assemblies also represent even difficult-to-align regions such as transposable elements. We ran RepeatMasker^35^ on several human ancestors, focusing on the recently-emerged L1PA6 family of L1 retrotransposons. When ascending the primate tree, approaching the origin of modern L1PA6 elements above the human–rhesus ancestor, L1PA6 elements appear increasingly similar to their consensus sequence (Extended Data Fig. 3b, Supplementary Fig. 6).

Despite its scale, sub-alignments of the 600-way are similar to smaller alignments of the same species. Within the 7.1 billion aligned base pairs involving human, mouse, rat or dog within the 600-way, 76.49% were present in an alignment with less than a tenth the number of species (Supplementary Fig. 7)—this similarity is in line with that observed between different alignments of these same species^14^. As expected, the alignments more strongly agree in functional regions, such as coding exons, than for the genome as a whole (Supplementary Fig. 8). The size and fraction of functional elements reconstructed in ancestors shared between the 600-way and smaller alignments of mammals and, separately, avians were also highly similar (Supplementary Figs. 9, 10).

To evaluate the relative accuracy of the progressive alignment process back to the amniote ancestor, human protein-coding transcripts and genes were mapped to the chicken genome using translated BLAT^36^, translated BLAST^37^, LASTZ^24^ and the 600-way alignment. Of 84,001 transcripts, BLAT mapped 70%, BLAST mapped 80%, LASTZ mapped 67%, and Progressive Cactus mapped 74%. Both Progressive Cactus and LASTZ had much lower levels of multi-mapping (2–3% of transcripts) than either translated method (16–51%) (Supplementary Tables 6–8). Comparison of Cactus and LASTZ coding sequence mappings to the union of the translated alignments, both in terms of individual gene counts and coding and mRNA bases, showed that Cactus has a marginally higher fraction of shared elements with the translated alignments than LASTZ (Supplementary Table 9). Supporting this result, comparing the median per-transcript and per-gene base-level Jaccard similarity of these mappings to chicken, while Progressive Cactus and LASTZ were most similar, Progressive Cactus was more similar to translated BLAT and Blast than LASTZ was (Supplementary Figs. 11, 12, Supplementary Table 10). Both Progressive Cactus and LASTZ have higher base-level similarity with existing chicken annotations than either translated alignment method (Supplementary Table 11).

**Fig. 4:**
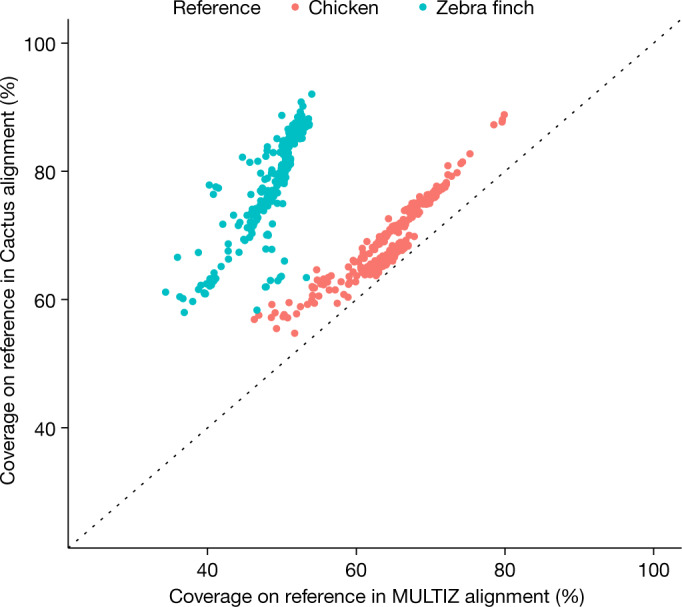
Comparing Cactus and MULTIZ alignment coverage. A comparison of coverage in the Progressive Cactus avian alignment compared to a chicken-referenced MULTIZ^22^ alignment of the same genomes. Coverage of both alignments on chicken and zebra finch is shown to illustrate the effects of reference bias on the completeness of the MULTIZ alignment. The diagonal dotted line indicates a slope of 1 (that is, if the coverage of MULTIZ and Progressive Cactus were equal). Source data

The B10K species were also separately aligned with MULTIZ^22^ using the chicken genome as the reference, allowing us to make a comparison between the two resulting alignments. Progressive Cactus aligned more total bases to chicken (covering an average of 69.4% of the chicken genome from the other species) than MULTIZ (64.9%), for an average increase of 47 Mb. Because, unlike Progressive Cactus, MULTIZ is reference-biased, the difference is starker when looking at the number of bases aligned to a genome not used as the MULTIZ reference (an average of 79% of the zebra finch covered versus 49.2%, for an average increase of 367 Mb) (Fig. 4).

## Discussion

The Vertebrate Genomes Project^38^ led by the Genome 10K^39^ and the Earth BioGenome Project^40^, among others, aim to release thousands of new, high-quality genome assemblies over the next decade. These projects will give us incredible insight into natural history, but will need massive genome alignments. We have shown that Progressive Cactus can create reference-free alignments of hundreds of vertebrate genomes efficiently. The B10K^32^ and Zoonomia^31^ consortia are using this alignment for comparative analysis, for example, analysing patterns of selection in unprecedented detail.

We focus on creating a reference-free alignment and ancestral reconstruction, allowing analysis of genome evolution throughout the entire tree rather than in comparison to one anointed reference. As the average assembly becomes ever more complete and accurate^38^, the value of such a reference-free approach grows. Similarly driven by technology improvements, sequencing efforts will increasingly produce multiple, phased de novo assemblies from different individuals in a population^41^. Progressive Cactus has already proved useful for comparison between assemblies of the same species^20^. Alignments of such assemblies are essential for annotation^9^ and variant characterization^42^ and should prove useful for reference-free pangenome construction of the variation present in a population^43^.

## Methods

### Data reporting

Sample selection was made according to the needs of the Zoonomia and B10K projects. The experiments were not randomized, and there was no blinding.

### Evaluation on simulated data

Twenty primate genomes were simulated using Evolver, managed using the evolverSimControl (https://github.com/dentearl/evolverSimControl, commit b3236deb) pipeline. The root genome used was derived from 30 megabases selected from the hg19 genome, and is available at http://courtyard.gi.ucsc.edu/~jcarmstr/datastore/progressiveCactusEvolverSim.tar.gz along with the Evolver configuration files that were used. The species tree used for the simulation was obtained from a catarrhine subtree of the 100-way alignment tree available on the UCSC browser.

The tree used was, in Newick format:


(((((((Human:0.00655,Chimp:0.00684)anc0e:0.00122,Bonobo: 0.00784)anc1e:0.003,Gorilla:0.008964)anc2e:0.009693,Orangutan:0.01894)anc3e:0.003471,Gibbon:0.02227)anc4e:0.01204,(((((Rhesus:0.004991,Crab_eating_macaque:0.005991)anc5e:0.001,Sooty_mangabey:0.001)anc6e:0.005,Baboon:0.003042)anc7e:0.01061,(Green_monkey:0.027,Drill:0.03)anc8e:0.002)anc9e:0.003,((Proboscis_monkey:0.0007,Angolan_colobus:0.0008)anc10e:0.005,(Golden_snub-nosed_monkey:0.0007,Black_snub_nosed_monkey:0.0008)anc11e:0.004)anc12e:0.009)anc13e:0.02)anc14e:0.02183,(((Marmoset:0.03,Squirrel_monkey:0.01035)anc15e:0.01065,White-faced_sapajou:0.009)anc16e:0.01,Nancy_Mas_night_monkey:0.01)anc17e:0.01)anc18e;


The alignments were generated using Progressive Cactus commit 51eb980b. The input files (the simulated genomes, input files and Progressive Cactus configuration file) are available at http://courtyard.gi.ucsc.edu/~jcarmstr/datastore/progressiveCactus.EvolverSim.CactusInput.EvenlySpread.tar.gz. A non-default configuration (included in the dataset) was used to change the alignment filtering in both runs to better support the high degree of polytomy in the star-tree runs. Four sets of 2, 6, 10 and 20 genomes were used, each of which were run three times to generate runtime estimates. The sets are as follows: 2 species: rhesus and marmoset; 6 species: rhesus, marmoset, gorilla, drill and black snub-nosed monkey, white-faced sapajou; 10 species: species from 6-species alignment and human, sooty mangabey, proboscis Monkey and Nancy Ma’s night monkey; 20 species: all species.

The runtime statistics were gathered using the toil stats command (the overall clock time was used, which represents central processing unit (CPU) time spent across all jobs). To generate the recall and precision statistics, multiple alignment format files (MAFs) were exported for each run (using hal2maf from the HAL^27^ package (https://github.com/ComparativeGenomicsToolkit/hal, commit 68db41d) with the --onlyOrthologs option using the rhesus genome as a reference) and compared with the Evolver MAF using mafComparator (https://github.com/dentearl/mafTools, commit 82077ac3).

### Comparison using Alignathon data

For comparison against other genome alignment methods, we aligned data (both the simulated ‘primates’ and ‘mammals’ datasets) used in the Alignathon using Progressive Cactus. For comparison, we downloaded all the original Alignathon entries in MAF format. We used the original Alignathon analysis workflow (https://github.com/dentearl/mwgAlignAnalysis, commit df98753) to reanalyse the MAFs, with the output of the newest Progressive Cactus version added, to generate the precision/recall statistics (which we extracted from the comparison against the most recent common ancestor (MRCA) truth set). The simulated-mammal results are shown in Supplementary Table 1, and the simulated-primates results are shown in Supplementary Table 2.

### Evaluation of the effect of the guide tree

The guide-tree analysis was performed on a set of 48 bird genomes previously published^29^. To reduce the amount of alignment work required, we subset these genomes down to the size of only a single chromosome, chicken chromosome 1 (by removing any contig or scaffold that had less than 20% of its sequence alignable to chicken chromosome 1). We used Progressive Cactus commit 36304707 for all alignments in this analysis.

The Prum and Jarvis topologies were adapted from Prum et al.^28^ and Jarvis et al.^29^, respectively. The ‘permuted’ topology was generated starting from the Jarvis topology, via three randomly chosen subtree-prune-regraft operations followed by three random nearest-neighbour-interchange operations. Each of these three topologies had branch-length estimates performed using phyloFit from the PHAST package (https://github.com/CshlSiepelLab/phast, commit 52e8de9) based on fourfold-degenerate sites of BUSCO orthologues. Finally, the ‘consensus’ tree was produced as a strict consensus of the Jarvis and Prum trees (collapsing all groupings that were not the same in both trees) using the ape::consensus method from the APE R package^44^. The branch-lengths for this tree were generated from the fitted branch lengths for the two input trees, using the consensus.edges function of the phytools R package^45^. The four final trees that were used in the four Progressive Cactus alignments are shown in Supplementary Fig. 1, and available in supplementary data in Newick format.

We further focused on the alignments with guide trees based on Jarvis^29^ and Prum^28^ (Supplementary Fig. 3) to establish what alignment differences resulted from different phylogenetic hypotheses. Supplementary Fig. 2 shows a refinement of the overall alignment-to-alignment *F*_1_ scores shown in Extended Data Table 2, showing the *F*_1_ scores for each species pair between the Jarvis- and Prum-based alignments. Each pair of species has an *F*_1_ score between Jarvis- and Prum-based alignments of at least 0.955.

### Effect of assembly quality on alignment quality

We aligned two sets of 11 boreoeutherian genomes: one in which 7 of the species were represented by relatively low-quality assemblies, and another in which the same 7 species were represented by higher-quality assemblies; the assemblies used are listed in Supplementary Table 4. The remaining four genomes had the same assemblies in both alignments to facilitate comparison (human, hg38; mouse, mm10; rat, rn6; and dog, canFam3). We used Progressive Cactus commit 36304707 for all alignments in this analysis.

### Generation of the 600-way alignment

The Zoonomia alignment was composed of two sets of mammalian genomes: newly assembled DISCOVAR assemblies and GenBank assemblies. The DISCOVAR genomes were masked with RepeatMasker commit 2d947604, using Repbase^46^ version 20170127 as the repeat library and CrossMatch as the alignment engine. The pipeline used is available at https://github.com/joelarmstrong/repeatMaskerPipeline, commit a6ad966. The guide-tree topology was taken from the TimeTree database^47^ (using release current in October 2018), and the branch lengths were estimated using the least-squares-fit mode of PHYLIP (http://evolution.genetics.washington.edu/phylip/getme-new1.html, version 3.695)^48^. The distance matrix used was largely based on distances from the 4d site trees from the UCSC browser^49^. To add those species not present in the UCSC tree, approximate distances estimated by Mash (https://github.com/marbl/Mash, commit 541971b)^49^ to the closest UCSC species were added to the distance between the two closest UCSC species. We used the hal package to process the HAL file (https://github.com/ComparativeGenomicsToolkit/hal, commit 68db41d).

The final guide tree is embedded in the HAL file, and available using the halStats --tree command. The 363 assemblies in the B10K alignment comprised four sets: 236 newly sequenced species for the ‘family’ phase of the project, assembled using SOAPdenovo2 and AllpathsLG, 42 assemblies already sequenced from the ‘order’ phase of the project, 36 assemblies taken from GenBank, and 49 assemblies contributed by other research groups. For the avian guide-tree, we used a tree that the B10K consortium derived as preliminary data from ultraconserved elements.

Both alignments were run on the AWS cloud over the course of 3 weeks for the avians and 2 months for the mammals, using a maximum of 240 c3.8xlarge instances and 20 r3.8xlarge instances. Because Toil’s autoscaling mode was used, this capacity was only fully used during the initial phase of the alignment, when the potential for parallelism was at its highest.

The 600-way alignment was formed by aligning the two roots of the B10K and Zoonomia alignments, using the xenTro9 (frog), latCha1 (coelacanth), and danRer11 (zebrafish) assemblies as outgroups. This created a ‘linker’ alignment connecting the roots of the two alignments. The B10K and Zoonomia alignments were then added to this linker alignment using the halAppendSubtree command.

### Micro-indel events within the 600-way

We extracted all insertion and deletion events by running the halBranchMutations (https://github.com/ComparativeGenomicsToolkit/hal, commit 68db41d) tool on every branch in the 600-way alignment. The ungapped insertion and deletion calls (represented by ‘I’ and ‘D’, respectively, within the output file) were filtered so that only calls spanning less than 20 bp (in the child for insertions, and the parent for deletions) were counted. The rate for each branch was then obtained by dividing the count of these micro-indel events by the total amount of sequence present in the child.

### Repetitive elements within ancestral sequences

We ran RepeatMasker (https://github.com/rmhubley/RepeatMasker, commit 2d947604) on all ancestral assemblies of human within the 600-way alignment (using RepBase^46^ version 20170127, selecting the ‘primate’ repeat library and choosing CrossMatch as the alignment engine). We also ran the same pipeline against human (as existing annotations used the ‘Homo_sapiens’ repeat library). All ancestors up to human-rhesus had over 78% of the human complement of L1PA6 elements (Supplementary Fig. 10).

### Human/chicken transcript alignment protocols

Protein-coding transcript annotations were obtained from the UCSC Genome browser^48^ tables. Human annotations are GENCODE V34 on hg38 (GRCh38/GCA_000001405.27) and chicken annotations are Ensembl 85 on galGal4 (GCA_000002315.2). Predicted RNA sequences for each protein-coding transcript are extracted from the genome. Only gene annotations on the primary assemblies were used, those on alternate loci, patches, and assembled sequences were dropped. This results in 84,001 transcripts in 19,695 genes for human and 15,328 transcripts in 14,499 genes for chicken. The human transcripts were then mapped from the human genome to the chicken genome. The steps for each method are outlined below, although the actual execution was done by partitioning the data and using a cluster. Command-line tools from the UCSC Genome Browser group and programs used came from: https://github.com/ucscGenomeBrowser/kent, commit 8a8d921, https://github.com/ComparativeGenomicsToolkit/hal, commit 68db41d, ftp://ftp.ncbi.nlm.nih.gov/blast/executables/blast+/2.10.0/, version tblastn: 2.10.0, and https://github.com/lastz/lastz, version 1.03.54.

### BLATX transcript alignment protocol

The BLATX alignments were created using protein-translated mode to align the mRNAs to the target genome with BLAT version 36x5. They were then filtered following the same protocol the UCSC Genome Browser uses for creating the other species RefSeq alignments:


blat -noHead -q=rnax -t=dnax -mask=lower <dest-genome.2bit> \



<src-rna.fa> <dest-rna-raw.psl>


We then filter to get near-best in genome. Alignment to chicken uses near best filter of -localNearBest = 0.010 while to human it uses -globalNearBest = 0.010:


faPolyASizes <src-rna.fa> <src-rna.polya>



pslCDnaFilter <nearBestOption> -minId=0.35 -minCover=0.15 -minQSize=20 \



-ignoreIntrons -repsAsMatch -ignoreNs -bestOverlap \



-polyASizes=<src-rna.polya> <dest-rna-raw.psl> <dest-rna-mapped.psl>


The transMapPslToGenePred command is then used to project the original coding sequence (CDS) onto the alignment.

### TBLASTX transcript alignment protocol

The TBLASTX alignments were created using the protein-translated ‘tblastx’ program to align the mRNAs to the target genome with BLAST+ version 2.10.0+.

The database is created using the repeat masking from the UCSC Genome Browser genomes to match what is used within the BLATX methodology above:


convert2blastmask -in <dest-genome.fa> -masking_algorithm repeat \



-masking_options “repeatmasker, default” -outfmt maskinfo_asn1_bin \



-out <dest-genome.mask>



makeblastdb -dbtype nucl -in <dest-genome.fa> -mask_data <dest-genome.mask>


The mRNAs are aligned and the resulting XML converted to PSL format, filtering to an e-value threshold of 0.01. These are then chained using a program the UCSC group developed for chaining BLAST alignments:


tblastx -db <dest-genome.fa> -db_soft_mask 40 -outfmt 5 -query <src-rna.fa> \



-out <dest-rna-raw.xml>



blastXmlToPsl -eVal=0.01 <dest-rna-raw.xml> <dest-rna-raw.psl>



simpleChain -outPsl -maxGap=75000 <dest-rna-raw.psl> <dest-rna-chained.psl>


The alignments produced are then filtered in the same manner as the BLATX alignments.

### LASTZ transcript alignment protocol

Both the LASTZ and Cactus transcript mappings use the ‘TransMap’^50^ projection alignment algorithm to project transcript annotation between genomes. The LASTZ alignment chains and nets^50–52^ were obtained from the UCSC Genome Browser downloads. These were then filtered to produce a set of syntenic mapping chains using these steps:


netFilter -syn <genomes.net> <syntenic.net>



netChainSubset -wholeChains <syntenic.net> <genome.chain> <mapping.chain>


### Cactus transcript alignment protocol

The Cactus alignments are extracted for all primary chromosomes from the HAL file and chained using the same chaining algorithm as the LASTZ chains, with the –noDupes option having a similar effect as the syntenic net filtering:


halLiftover --outPSL --noDupes 600way.hal <srcOrganism> \



<srcChroms.bed> <destOrganism> <src-dest.psl> <genome.psl>



axtChain -psl -linearGap=loose -scoreScheme=HoxD55.q <genome.psl> <mapping.chain>


The ‘TransMap’ protocol is used for both the LASTZ and Cactus mapping chains to produce alignments of the transcripts to the other genomes. This used the ‘pslMap’ command to do the mapping and ‘pslRecalcMatch’ to update the statistic in the alignments:


pslMap -chainMapFile <src-rna.psl> <mapping.chains> <dest-rna-over.psl>



pslRecalcMatch <dest-rna-over.psl> <dest-genome.2bit> <src-rna.fa> <dest-rna-raw.psl>


The alignments produced are then filtered in the same manner as the LASTZ alignments.

### Transcript and gene alignment subsets and comparison

To facilitate the comparative analysis of the alignment methods, we created reduced sets of the alignments using two different approaches. Although both BLATX and TBLASTX will align UTR, the strength of protein-translated methods is in recognizing distant coding sequence relationships. Alignment projection-mapping methods were previously shown^53^ to align more UTR bases than translated methods. To facilitate comparisons, CDS alignments from each method were created by trimming the RNA alignments to contain only the CDS regions as defined by the human annotation set.

Although mapping all transcripts is useful, particularly for understanding the utility of the methods in assisting genome annotation, individual transcripts overlap, biasing assessment of transcribed mappings to genes with larger transcript numbers. To remove most of this base multiplicity from comparisons, in addition to showing full transcript results, subsets of the alignments are created using only one representative transcript per gene. For the full RNA alignments, the longest RNA for each gene was chosen, with the CDS alignments choosing the transcript with the longest CDS. The biology of overlapping gene structures and the ambiguities in defining genes cause around 4% of genomic bases to appear in more than one gene in the RNA, and 3% in the CDS gene sets owing to overlap.

Individual pairwise alignments were compared at the base-level, consistent with the earlier comparisons reported. In brief, alignment similarity is computed by comparing the set of shared aligned pairs. That is, a pairwise alignment can be viewed as a set of aligned base pairs, each a coordinate from the source (human) and target (chicken) genome. The Jaccard index is, in this context, the number of aligned pairs identical between the two alignments divided by the union of all aligned pairs in the two alignments. It is worth noting that translated alignments are encoded for comparison using their induced base-level alignments. Transcripts or genes that are not aligned by either of the aligners being compared are assigned Jaccard indices of zero.

To account for human bases that map to multiple bases in chicken (which occurs frequently for the translated alignment methods that include very distant, fragmented, paralogous alignments, but much less often for the non-translated methods), when comparing the alignments of an mRNA or CDS between two methods, if either or both methods produces multiple alignments, we pick the pair of mappings (one from each method) with highest shared similarity to report. This generally has the effect of removing distant paralogues from the comparison.

### Progressive Cactus methods

Progressive Cactus builds upon the original Cactus program, in particular the CAF and BAR algorithms, which are described in detail in the original publication. In overview, the CAF algorithm (short for Cactus Alignment Filter) is an algorithm designed to construct a sequence graph from an input set of local alignments (in the Progressive Cactus pipeline computed using LASTZ). We omit a complete definition here, but a sequence graph represents the alignment of a set of nucleotide strings. It can formally be represented using a bi-directed or bi-edged graph^54–56^ (Supplementary Fig. 13a). Larger nucleotide strings are encoded as walks through sequence graphs (Supplementary Fig. 13b); in the bi-edged representation an alignment between two or more substrings is represented by both strings visiting a common sequence edge; in Progressive Cactus each sequence edge represents an alignment ‘block’, a set of oriented substrings in the set of input strings which are considered to be gaplessly aligned. A key property of alignments represented by sequence graphs is that the alignments they represent are equivalence relations: that is, alignments are transitive, reflexive and symmetric. The core challenge the CAF algorithm addresses is sub-selecting which local alignments from the input set to include in the sequence graph, because typically a collection of local alignments computed with a tool like LASTZ will contain numerous transitive inconsistencies which when combined will create implausible, high degree alignment blocks in the sequence graph. The CAF algorithm uses the 3-edge connected components of a sequence graph to define a restricted form of cactus graph such that there exists a homomorphism from the alignment blocks in the sequence graph onto the resulting cactus graph (Supplementary Fig. 13c). In the constructed cactus graph each edge is a member of exactly one simple cycle. These simple cycles correspond to ‘chains’ of alignment blocks, maximal sequences of blocks whose aligned substrings appear in the same order and orientation in the input strings. The CAF algorithm iteratively filters the input set of alignments to remove local alignments that create short simple cycles in the cactus graph, this is achieved by deleting alignment blocks from the sequence graph involved with these short cycles. The result of the CAF algorithm is a filtered set of local alignments represented using a sequence graph. To add to the output sequence graph of the CAF algorithm the BAR algorithm constructs a detailed alignment by extending gapped alignments from the ends of each alignment block, using a greedy approach to force consistency between the alignments constructed starting from connected alignment blocks. In Progressive Cactus the CAF and BAR algorithms are applied to create an alignment of the corresponding set of in-group and out-group species for each internal node of a guide tree.

Below we provide updates on the changes made to Cactus to create Progressive Cactus.

### Preliminary repeat-masking

Progressive Cactus requires input genomes to be soft-masked, but often repetitive sequence goes unmasked due to poor masking or incomplete repeat libraries for newly-sequenced species. This can negatively affect alignment runtimes (as alignments need to be enumerated to and from all copies of a repetitive sequence) and impact quality. For this reason, we mask overabundant sequence before alignment, using a strategy not based on alignment to repeat consensus libraries, but on over-representation of alignments. We first divide each genome into small, mutually overlapping chunks. For each chunk, we align it to itself and a configurable amount of other randomly sampled chunks (currently 20% of the total pool). To avoid combinatorial explosion due to unmasked repetitive sequence, we use a special mode of LASTZ which stops exploring alignments from any region early if a maximum depth is reached (using the flag --queryhsplimit=keep,nowarn:1500, which stops after a high-scoring-pair depth of 1,500). We then soft-mask any region covered by more than a configurable number of these alignments (currently set to 50). Further details can be found in the src/cactus/preprocessor section of the Progressive Cactus codebase. Although the preprocessing step is automatically run as part of the pipeline, we also provide a cactus_preprocessor utility to run only the preprocessor without producing a full genome alignment.

### Local alignment and outgroup selection

The alignment process for each subproblem begins with a series of local alignments generated using LASTZ. The local alignments fall into two sets: a set of all-against-all alignments among the ingroup genomes, and a set of alignments from ingroup genomes to outgroup genomes. We have found outgroup selection to be absolutely crucial in creating an acceptable ancestral reconstruction: any missing data or misassembly in the outgroup that causes a true deletion in one of the ingroups to be misinterpreted as an insertion in others will mean that the ancestor contains less sequence than it ought to. This missing sequence in turn impacts the alignment between the entire subtree below the reconstructed ancestor and the entire supertree above it: the missing sequence will never be aligned between the subtree and supertree. To avoid this we attempt to use multiple outgroup genomes in each subproblem (by default, the three nearest outgroup genomes, as measured by branch-length). Naively aligning each ingroup against multiple outgroups would significantly increase the computation time; to avoid this we note that in general any region already containing an outgroup alignment benefits very little from aligning an additional outgroup. Therefore, we iteratively align each ingroup against one outgroup at a time, pruning away any ingroup sequence already covered by the previous outgroup alignments. In this way, the computational cost is reduced to be far less than naively aligning against the entire outgroup set, while still retaining nearly all of the benefit. In addition, we allow the user to designate certain genomes in the input as being of particularly high quality; these are chosen as outgroups if possible to avoid problems with missing data in regions such as mitochondrial or sex chromosomes that are often missing from some assemblies but not others.

### Paralogy resolution

Users of a genome alignment are often interested in ‘orthology’ relationships, rather than all ‘homology’ relationships, between a set of sequences. For example, when comparing human and chimpanzee *KZNF* genes, providing an alignment from each gene to the over-400^56^ homologous *KZNF* genes in the other genome is nigh-useless; the user is likely interested in only the orthologous copy or copies (in the case of a lineage-specific duplication) in the other genome. For this reason, Progressive Cactus alignments are capable of representing complex orthology/paralogy relationships, with an ability to display the alignment(s) labelled as orthologous, but also the option for a user to request alignments to paralogues at a customizable divergence-time threshold. This is achieved by implicitly producing a gene tree as the alignment is built, albeit with some restrictions, namely that a duplication event is represented by multiple regions in the child(ren) aligned to a single region in the parent species. This forbids the representation of gene-tree-species-tree discordance as would occur in incomplete lineage-sorting or horizontal transfer, as well as the exact ordering of multiple duplication events along a single branch. The restricted problem we solve at each subproblem step is that each alignment block should represent all regions orthologous to a single region of the ancestral sequence, possibly multiple per species; we make no attempt to fully resolve the gene tree when multiple duplications take place along a single branch. However, this still requires resolving the timing of all duplication events to the lineages of the tree: duplicated sequences whose coalescence precedes the speciation event represented in the subproblem should be split, while those following the speciation event should be kept together.

To achieve the desired alignment blocks in each subproblem, in constructing the initial sequence graph during the CAF algorithm Progressive Cactus greedily chooses which pairwise alignments to include in an effort to prevent paralogous alignments between the ingroup species. We developed two algorithms. Both are greedy algorithms designed to rank the pairwise local alignments and then iteratively add the alignments to the graph, at each step choosing to accept or reject the addition of alignments to the graph. Each added alignment ‘glues’ together two alignment blocks, splitting existing alignment blocks as necessary and merging the resulting two alignment blocks into one new block in the graph (Supplementary Fig. 14).

The first algorithm, which was used in previous, beta versions of Progressive Cactus, relied on an outgroup-based heuristic to resolve duplication timing. This heuristic, which we term ‘single-copy outgroup filtering’, first sorts all the LASTZ alignments by their score in descending order. Then, starting from the highest-scoring alignment, it iteratively adds one alignment at a time to the sequence graph, rejecting the gluing of any two blocks if the resulting alignment block would contain two or more substrings from the same outgroup genome. In this way the heuristic refuses to glue blocks when the resulting block would contain homologies that imply duplications in the outgroups. These self-homologies within the outgroup would necessarily involve duplication events that occurred above or outside of the subtree rooted at the MRCA of the ingroup genomes. Since the goal at each progressive step is to determine (the transitive closure of) orthology relationships within this subtree, refusing these outgroup self-homologies proves useful for assigning orthology between ingroups. Unfortunately, this method is very sensitive to incomplete outgroup assemblies (containing an incorrect number of copies of a duplicated region) or variation in the similarity between closely related paralogues, causing assignment to the wrong copy. As seen in Extended Data Fig. 1, this filtering method tended to resolve duplications far too early, often causing paralogues to be called orthologues.

To remedy this problem, we developed an improved duplication-timing method, which we term ‘best-hit filtering’. The method preprocesses the local alignments to define for every base in every input genome a ranking by score of the local alignments that overlap it. The sequence graph is then built by first including the highest-scoring alignment for each base in each genome. We refer to this highest-scoring set as the set of ‘primary’ alignments and the remaining alignments the ‘secondary’ alignments. Note this definition is asymmetric: a pairwise alignment may be primary for one of the substrings it aligns and secondary for the other. All primary alignments are added to the initial graph unconditionally because they represent the most likely orthologue relationship (or in the case of multiple orthology, probably a random orthologue) (Supplementary Fig. 15). The set of primary alignments represents a conservative set of alignment relationships that should include nearly no alignments to ancient paralogues. However, in regions that have undergone many rounds of lineage-specific duplications (which should all be aligned together in the restricted duplication-timing problem we described above), the set of primary alignments will often by chance not align all copies together. For this reason, after adding the primary alignments we iteratively add secondary alignments, going in descending order of score, rejecting any secondary alignment that would glue together any two existing blocks that both contain sequences from the same outgroup species (similar to the ‘single-copy outgroup filtering’ method)—this allows lineage-specific duplications of ingroup genomes to correctly land in the same block, while avoiding merging blocks from likely-paralogous alignments.

Of the two methods, the newer best-hit filtering removes many more probably paralogous alignments, especially to closely related genomes, while leaving approximately the same amount of sequence covered by at least one alignment. For example, we ran two versions of Progressive Cactus, one using the best-hit filtering and one using the outgroup filtering (commits 450da74 [best-hit filtering] and aca859f [outgroup filtering]), using the following tree:


(((((Human:0.006969,Chimp:0.009727):0.025291,Rhesus:0.044568):0.07,Tree_shrew:0.19):0.03,(Kangaroo_rat:0.17,(Mouse:0.072818,Rat:0.081244):0.11):0.150342):0.02326,((Dog:0.07,Cat:0.07):0.087381,((Pig:0.06,Cow:0.06):0.104728,Horse:0.05):0.05):0.04);


Comparing the best-hit filtering alignment and the one using the single copy outgroup filtering, the amount of human sequence mapping to two or more places in the chimpanzee genome was reduced from 6.1% to 2.6%, while the total amount of human covered by chimpanzee actually increased owing to the removal of ancient homologues, simplifying the initial alignment relationships (see Extended Data Fig. 1a, b for an example visualization and Extended Data Fig. 1c for aggregate statistics).

The alignment files are accessible in the URLs listed at Supplementary Table 12, and the assemblies used are listed in Supplementary Table 13. Coverage statistics from the resulting alignments were obtained using the halCoverage tool (https://github.com/ComparativeGenomicsToolkit/hal, commit 68db41d). To confirm that these improvements were likely caused by the removal of paralogous rather than orthologous alignments, we compared phylogenetic trees implicit in the columns of HAL alignments to independently re-estimated approximately maximum likelihood (ML) trees produced by FastTree (http://www.microbesonline.org/fasttree/, version 2.1.11)^57^ for the same regions. The duplication-timing evaluation was performed using a custom pipeline (https://github.com/joelarmstrong/treeBuildingEvaluation) designed to sample columns from a HAL file and evaluate their trees against an independently re-estimated tree of the same region. For this analysis we used the two 12-boreoeutherian alignments described above, sampling 10,000 columns from the human genome. The comparison trees were built from a context of 1,000 bases around the entries in each sampled column using FastTree 2.1.10 and the -gtr -nt options. Only duplicated columns were counted in the final output (columns containing no duplications did not count in the results). The coalescence pairs were evaluated using the --onlySelf option, meaning that only pairs that included the sampled site were counted in the results. To avoid weighting columns with a high number of copies per genome more than columns with a low number of copies per genome, only a single coalescence was randomly sampled per column.

Because HAL does not produce a fully binarized history of duplication events, we compared the MRCA of randomly selected pairs of sites from genomes containing a duplication within the column. If the MRCA species in the HAL tree is a descendant of the MRCA species within the reconciled ML tree, that implies that there are paralogues represented as orthologues within the HAL tree (since a duplication event must have been resolved too early). Similarly, if the MRCA species within the HAL tree is an ancestor of that within the reconciled ML tree, a duplication event must have been resolved too late in the HAL tree, indicating additional false loss or deletion events. The number of paralogous alignments (represented by the coalescence time between duplicated sequences being too ‘early’ in the HAL tree relative to the ML tree) in the alignment of the 12 boreoeutherian genomes was clearly reduced (46% in the outgroup filtering versus 26% in the best-hit filtering) (Extended Data Fig. 1d).

We separately ran the Comparative Annotation Toolkit (CAT; https://github.com/ComparativeGenomicsToolkit/hal, commit 68db41d)^9^ on identical human, chimpanzee and gorilla assemblies (hg38, panTro6, and gorGor5 assemblies) in two alignments using the outgroup and best-hit filtering methods. We ran using the GENCODE V30 gene set^58^. We projected the transcripts solely via transMap without the use of the AUGUSTUS modes. Multiple-mapping statistics and the gene composition of the final gene set were taken from the filter_tm_metrics.json file in the CAT output.

Not only was CAT less likely to identify a human gene in multiple chimp loci using the best-hit filtering (for example, 6.5% versus 9.8% multiple-mappings across all genes in chimp, and 5.9% versus 13.8% for the recently-duplicated KRAB zinc-finger gene family) (Extended Data Fig. 1e), but as a result orthologues for 104 more human genes were identified in the output gene set for chimp (182 in gorilla) (Supplementary Table 14). This is probably because tens of thousands of fewer paralogous transcripts were filtered out in the initial filtering phase of CAT (Supplementary Table 15), reducing confusion about which transcript projection to put into the gene set.

### Removing recoverable chains

The original CAF algorithm was focused on removing small rearrangements while retaining as much of the original alignment relationships as possible in the filtered cactus graph. However, because the input local alignments are insensitive, the original alignment relationships are likely to have missed certain homologies. This can result in what we term ‘incomplete blocks’: blocks that contain some alignment relationships but are missing others, that is, are proper subsets of the corresponding ‘true’ alignment block. In our anchor-and-extend process, once a block becomes an anchor it can never be modified. As a result, these incomplete blocks will remain incomplete: they prevent the true alignment relationship from being found, even if an adjacent syntenic anchor block is complete and contains all desired alignment relationships. These problematic incomplete blocks become more prevalent at longer evolutionary distances: the local aligner will miss more true homologies at increasing distances, causing more incomplete blocks and in turn a far worse alignment.

To remove these incomplete blocks, Progressive Cactus originally relied on a heuristic that identified blocks that were ‘likely’ to be incomplete, removing blocks that did not have alignment relationships between all ingroups. However, this heuristic performed poorly in the presence of deletions or missing data: any large deletion in one ingroup could cause huge stretches of the other ingroup(s) to be left unaligned. To remedy this, we have developed a new alteration to the CAF algorithm, one that now focuses on maximizing the potential size of the alignment graph ‘after’ extension as opposed to ‘before’ extension. We call this addition ‘removing recoverable chains’ because it identifies chains in the cactus graph that represent alignments that could be recovered by the BAR algorithm extension process.

The algorithm is applied as a post-processing step after the CAF process, which proceeds as normal. After the cactus graph is created and filtered, the algorithm identifies ‘recoverable blocks’. Each block is composed of segments, each of which represents a non-overlapping region of a sequence and which strand is being aligned; we briefly review the necessary terminology, but see^59^ for additional context. We call a segment ‘*a* left-adjacent’ to another segment ‘*b*’ if ‘*a*’ represents the positive strand and ‘*b*’ comes before ‘*a*’ in their sequence and there is no other segment between them. Similarly, we call a left-adjacent to *b* if *a* is on the negative strand and a comes before b in their sequence ordering with no other intervening segment. If *a* is left-adjacent to *b*, then *b* is right-adjacent to *a*.

A block is called ‘recoverable’ if, in the case that the block was removed, all its regions would be contained entirely within a single end alignment in the BAR extension phase. The end alignments are identified by looking at all unaligned sequences between the adjacent segments of a single ‘end’ of a block: in short, two end alignments are created for every block, one for all sequences between each segment and its left-adjacent segment, and similarly for the right-adjacent segments. In practice, this means that for some block *A*, it is recoverable if all its segments are all left- or right-adjacent to segments from the same block *B* ≠ *A*.

Whether a block is recoverable depends only on its immediate neighbouring blocks. However, it is interesting to consider the maximum set of recoverable blocks, and, by contrast, of unrecoverable blocks—these unrecoverable blocks represent a minimal set of anchors that can be extended from to recover the alignment relationships from the original sequence graph as well as potential additional alignment relationships.

Because the chains and nets within the cactus graph represent a hierarchy of the rearrangements implicit in the alignment, they are helpful for finding a smaller set of anchors to extend from. We consider what anchors could provide recoverability to a block: if a block *A*’s segments would lie within the end alignment of *B* if all the recoverable blocks between *B* and *A*, including *A*, were destroyed, we call *A* recoverable given *B*. The relationship is transitive: if block *A* is recoverable given block *B*, and *B* is recoverable given *C*, then *A* is recoverable given *C*. All blocks in a chain are recoverable given each other, since all blocks in a chain are collinear with each other, potentially with intervening rearrangements located further down the chain/net hierarchy. Similarly, if any block in a chain is recoverable given another block above the chain in the chain/net hierarchy, the entire chain is recoverable given that block. Owing to this fact, to determine the recoverability status of all blocks, we only have to examine the blocks at the ends of chains and their immediate neighbours, rather than every block.

Although in principle we would need to keep only one block within even unrecoverable chains (since all other blocks within the chain would be recoverable given that single block), to save computational effort in realignment we only destroy or keep entire chains as a unit. In the same spirit, to avoid spending needless effort when the chain is recoverable but very likely is not incomplete, we apply a heuristic and do not remove chains that contain the same number of copies in all ingroups and outgroups.

After identifying and removing all recoverable blocks, some blocks previously marked unrecoverable may become recoverable (because adjacent blocks were removed). For this reason, we run the process of identifying and removing recoverable chains multiple times in a loop, until either no recoverable chains are identified or a limit on the number of cycles is reached. The structure of the cactus graph may change after removing recoverable blocks, so we recompute the cactus graph after every removal step. The process that is followed is described in pseudocode as follows:


**function** RemoveRecoverableChains(*G*, *n*)



**for** 1 ... n **do**



*cactusGraph* ← CreateCactusGraph(*G*)


*RecoverableChains ←* ∅


**for** chain *C* in *cactusGraph***do**



**if**


⊳ A single adjacent end offers the potential for recoverability


(|*C*.leftAdjacencies| = 1 **or** |*C*.rightAdjacencies| = 1)


⊳ Shared adjacencies indicate a non-recoverable rearrangement

***and C.leftAdjacencies ∩ C.rightAdjacencies = ***∅

⊳ Links between chain ends indicate a non-recoverable duplication


**and***C*.leftEnd ∉ *C*.rightAdjacencies **then**



*RecoverableChains* ← *RecoverableChains* ∪ {C}



end if



end for



**if** |RecoverableChains| = 0 **then**



break



else



Destroy each chain in *RecoverableChains*



**end if**



**end for**



**end function**


### Improvements from removing recoverable sequence

To quantify the effect that the process of removing recoverable chains (described above) had on real alignments, we ran alignments on a set of nine Euarchontoglires genomes with the feature turned on and off. The tree used was:


(((((((human:0.00877,gorilla:0.008964):0.009693,orang:0.01894):0.015511,rhesus:
0.037601):0.07392,tarsier:0.1114):0.034014,tree_shrew:0.19114):0.002,(kangaroo_rat:0.171759,(chinese_hamster:0.14,mouse:0.132282):0.11015):0.114051)euarchontoglires:0.020593,(cow:0.18908,dog:0.13303):0.032898);


We used Progressive Cactus commit 56874bde, with the --root euarchontoglires option so that cow and dog were used only as outgroups. Coverage on human increased for all genomes when recoverable chains were removed, especially for those most distant from human (Supplementary Fig. 16). This probably reflects the fact that though the losses caused by not removing recoverable chains in any single subproblem are relatively small, they can compound to be quite considerable in large alignments since many subproblems are involved in creating the alignment between distant species (such as human and mouse, which are separated by seven internal nodes in this tree).

### Ancestral genome reconstruction

The core of what makes the progressive alignment algorithm possible is the ancestral reconstruction generated in each subproblem. This assembly serves as a summary of each subproblem alignment; the alignable sequence between the genomes in the subtree below the ancestor, as well as that alignable between the subtree and the supertree above the ancestor, is all present in the ancestral reconstruction. The ancestral sequence contains a base for each column in all blocks which contain an alignment between two ingroups and/or an ingroup and an outgroup—any alignment purely between outgroups is discarded. The order and orientation of the blocks relative to one another is chosen via a previously published algorithm for ordering a pangenome^60^.

The identity of the ancestral bases is inferred via maximum-likelihood on a Jukes-Cantor model^61^ of evolution using Felsenstein’s pruning algorithm^62^ on the subtree of the guide tree induced by the genomes in the subproblem. These base-calls are generated as the alignment is being made, so they necessarily take only a part of the alignment information into account and may be different than the ideal base-calls would be if taking into account information across the entire alignment. However, we provide a tool, ancestorsML, distributed as part of the HAL toolkit, that re-estimates ancestral base-calls after completion of the alignment if desired.

### Adding a new genome to an existing alignment

Progressive Cactus supports adding a new genome to an existing alignment by taking advantage of the tree structure of the progressive alignments it produces. There are three ways that a new genome can be added to an alignment, depending on its phylogenetic position relative to the existing genomes: (1) as outgroup to all the existing genomes in the alignment; (2) by being added as a new child of an existing ancestral genome in the alignment; or (3) by splitting a branch in the existing alignment, creating a new internal node and two new branches (Extended Data Fig. 2). Progressive Cactus allows adding a new genome in any of these ways, though the details differ (as described below). Assemblies can be replaced with new versions by simply deleting them and adding the new assembly in as a leaf. Adding multiple genomes is possible, either iteratively or (if the new genomes are monophyletic) by aligning together the new genomes and adding in the ancestral clade root.

Adding a genome as an outgroup is straightforward because it follows the normal progressive process: the root of the existing alignment and the new genome can be aligned together into a supertree alignment in which the existing subtree alignment can be appended to. A genome can be added as a new child of an existing internal node by simply aligning the new child, its siblings, and its parent together, without inferring a new ancestral genome. Adding a genome by splitting an existing branch is the least straightforward, but is key if the topology of the alignment or the accuracy of the ancestral genomes is important. To add a genome to an existing alignment, two subproblems are required: one relating the new genome and its new sibling in the target tree, constructing the ancestral genome that will split the existing branch, and one relating this new ancestral genome, its sibling, and its parent.

After the addition of a new genome as an ingroup (by adding it to a node or a branch), at most a single ancestral sequence is re-inferred. This prevents any information from the new genome from propagating to the rest of the tree. Although this saves considerable effort in recomputing other parts of the alignment, it also means that, occasionally, rare stretches of sequence in a newly added genome would not be properly aligned to distant outgroups because they were deleted or missing in the new genome’s close relatives. Re-inferring the ancestral genomes on the path from newly added genomes to the root should address this issue if it appears.

We tested the effect of adding a new genome to an existing alignment using the same set of simulated catarrhine genomes as described above. To replicate the use-case of an end-user wanting to add a genome to a previously-created alignment, we generated an alignment holding out one of the 20 genomes (the crab-eating macaque), and added that genome back into the alignment by both splitting an existing branch (resulting in the same topology as a full alignment would), and by adding the macaque as a new child of an existing ancestor (creating a trifurcation which did not exist in the original tree. All alignments for this analysis were generated using Progressive Cactus commit 49e80082 and we used tools from the hal package (https://github.com/ComparativeGenomicsToolkit/hal, commit 68db41d).

To add the crab-eating macaque back in as the child of an existing node (the add-to-node strategy), we ran a single new alignment with the tree (Rhesus:0.006, Crab_eating_macaque:0.007, Sooty_mangabey:0.001)anc6e;. The anc6e genome from the original, held-out alignment was used as an unreconstructed ancestral input sequence. We set the ‘runMapQFiltering’ option in the config file to ‘0’ and the ‘alignmentFilter’ option to ‘singleCopyOutgroup’, because these options produce a better alignment of polytomies. We merged the resulting HAL file into a new copy of the existing alignment via the command:


halReplaceGenome <copy of held-out alignment> anc6e \



--topAlignmentFile <held-out alignment> \



--bottomAlignmentFile <add-to-node alignment>.


To add the macaque by splitting a branch (the add-to-branch strategy), we ran two separate alignments. We ran the first with the tree (((Rhesus:0.004991, Crab_eating_macaque:0.005991) anc5e:0.001, Sooty_mangabey:0.001)anc6e:0.005, Baboon:0.003042)anc7e; (with the --root anc5e option so that only a single subproblem was run), generating a newly reconstructed anc5e ancestor. We then ran a second alignment with the tree (anc5e:0.001, Sooty_mangabey:0.001)anc6e;, again providing the anc6e assembly from the original alignment rather than inferring a new reconstruction. (We note that these two subproblems could have been run in a single alignment invocation, resulting in the same amount of alignment work but a slightly more complicated merging process.) To merge these two add-to-branch intermediate alignments into a full alignment, we first removed the Rhesus genome from a new copy of the held-out alignment. We then ran  halAddToBranch <held-out alignment> <first add-to-branch alignment> <second add-to-branch alignment> anc6e anc5e  Rhesus Crab_eating_macaque 0.001 0.006.

Both methods resulted in alignments that had accuracy deviating less than 0.1% from the full alignment that included the macaque from the start: both addition methods, as well as the full alignment, achieved an *F*_1_ score of 0.926 (Extended Data Table 1). We evaluated the performance of these new alignments using mafComparator in the same way as described above. In the interest of narrowly determining the accuracy of alignments involving the newly added genome, we counted only aligned pairs involving the Crab_eating_macaque genome when calculating precision, recall, and *F*_1_ scores.

### Reporting summary

Further information on research design is available in the Nature Research Reporting Summary linked to this paper.

## Online content

Any methods, additional references, Nature Research reporting summaries, source data, extended data, supplementary information, acknowledgements, peer review information; details of author contributions and competing interests; and statements of data and code availability are available at 10.1038/s41586-020-2871-y.

### Supplementary information

Supplementary Information A merged PDF containing the individually numbered supplementary figures and supplementary tables referenced in the main text and methods. There are sixteen supplementary figures and fifteen supplementary tables. These supplementary items provide supporting information about the individual experiments described.

Reporting Summary

Supplementary Data Excel spreadsheet of mammalian genome assemblies used in the 600-way alignment.

Supplementary Data Excel spreadsheet of avian genome assemblies used in the 600-way alignment.

### Source data

Source Data Fig. 2

Source Data Fig. 3

Source Data Fig. 4

## Figures and Tables

**Extended Data Table 1:**
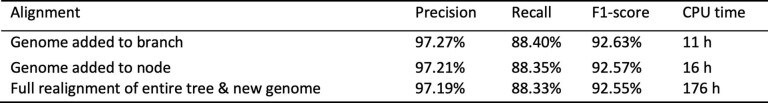
Adding a new genome to an alignment of simulated genomes

Precision, recall and *F*_1_ score statistics are all of the aligned pairs that contain a base of the added genome. An alignment in which the genome was included initially is shown for comparison.

**Extended Data Table 2:**
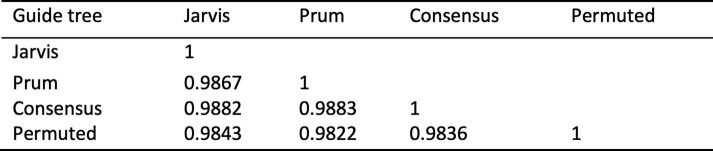
Alignment similarity between four alignments of the same 48 avian genomes with different guide trees

The similarity between each pair of alignments is represented by the *F*_1_ score of aligned-pair relationships in the two alignments.

## Data Availability

The 600-way genome alignment is composed of data gathered for the Zoonomia mammalian genomes project and data from the B10K project. All genomes have been archived in GenBank, spreadsheets containing all the accession numbers of the assemblies is provided in the Supplementary Information. The 600-way alignment is available in HAL format from https://cglgenomics.ucsc.edu/data/cactus/. At the same location we also provide the subset of the alignment containing the Zoonomia genomes, the subset of the alignment containing the B10K genomes, and a visualization of the alignments and associated data as an assembly hub for the UCSC Browser^49^. Note that the B10K consortium is organizing phylogenomic and other analyses with the avian alignment and sequencing data. We encourage people to contact us for collaboration if they are interested in using these data for phylogenetic analyses. Source data are provided with this paper.

## Code availability

The Progressive Cactus pipeline is available at https://github.com/ComparativeGenomicsToolkit/cactus under the MIT license, version 1.0 is archived here: 10.5281/zenodo.3873410. The exact version of Progressive Cactus used for each of the analyses described above varies; for the commit used in each analysis, see the appropriate section of the Methods.
