# Supplementary material for: Progressive Cactus is a multiple-genome aligner for the thousand-genome era
Source: Nature. 2020 Nov 11;587(7833):246–51. doi: 10.1038/s41586-020-2871-y (PMC7673649; doi:10.1038/s41586-020-2871-y)
Supplement: Supplementary file 1 — A merged PDF containing the individually numbered supplementary figures and supplementary tables referenced in the main text and methods. There are sixteen supplementary figures and fifteen supplementary tables. These supplementary items provide supporting information about the individual experiments described. [file 41586_2020_2871_MOESM1_ESM.pdf]

nature research

https://doi.org/10.1038/s41586-020-2871-y

Supplementary information

Progressive Cactus is a multiple-genome aligner for the thousand-genome era

In the format provided by the authors and unedited

Supplementary Information: Progressive Cactus: a multiple-genome aligner for the thousand-genome era

July 9, 2020

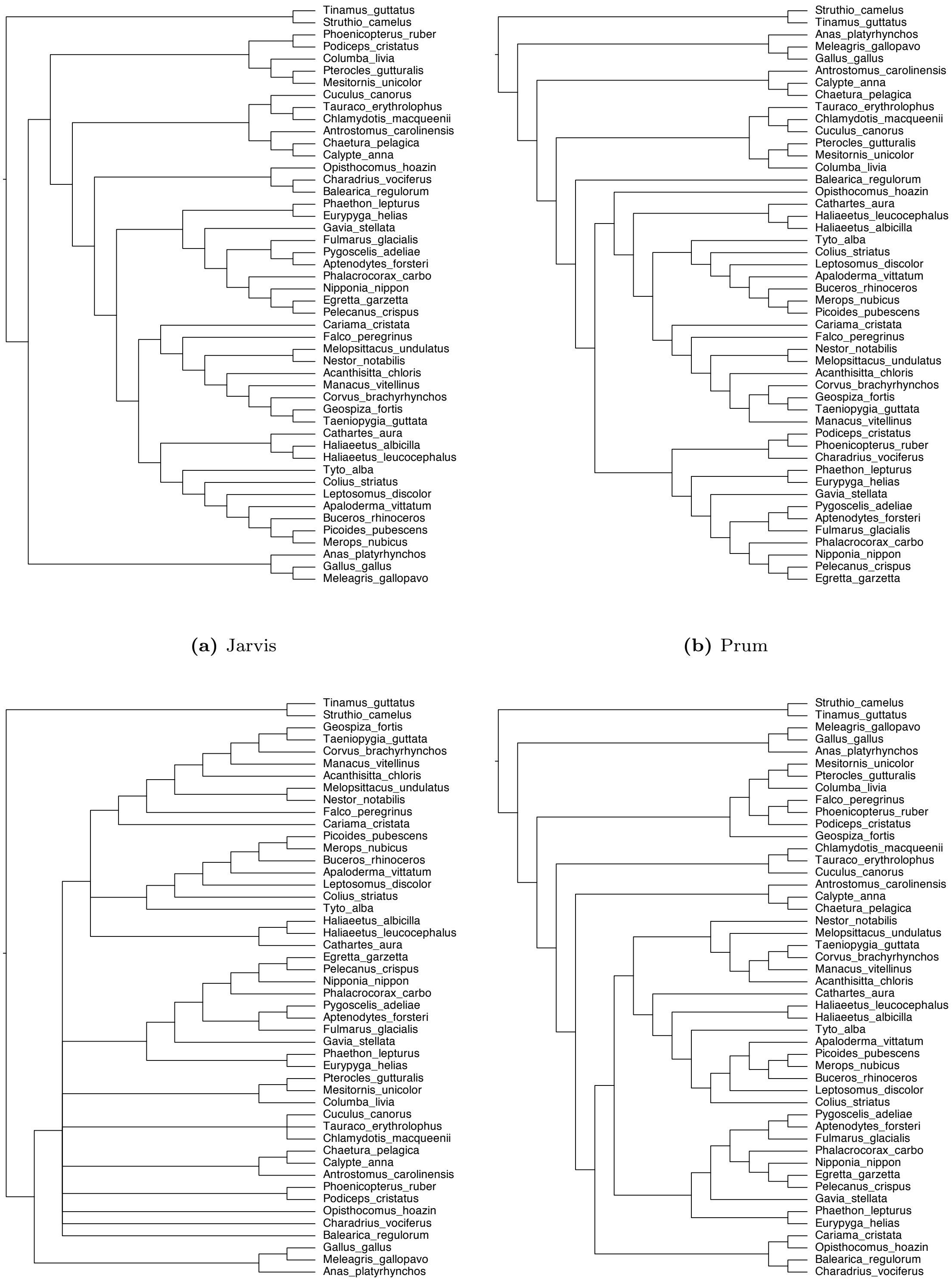

(a) Jarvis

(b) Prum

(c) Consensus

(d) Permuted

Supplementary Figure 1: Guide trees used in the guide-tree influence analysis.

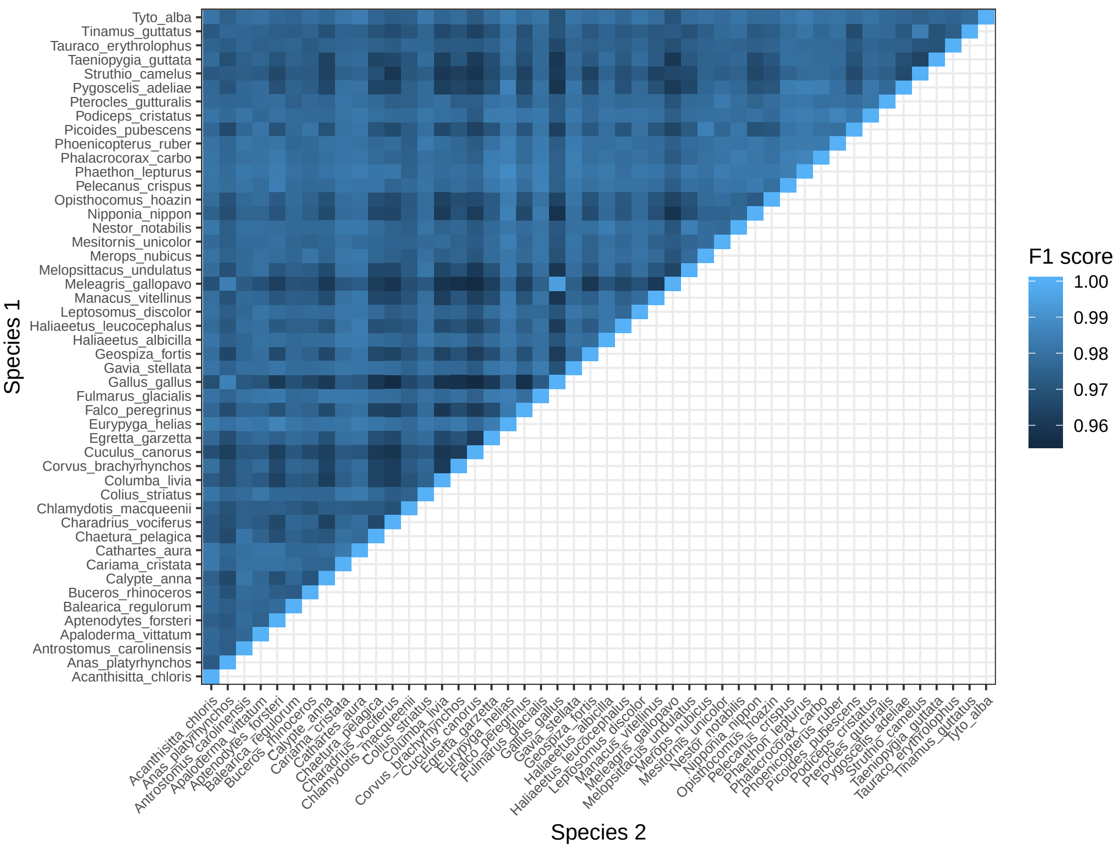

Supplementary Figure 2: Species-by-species breakdown of similarity between the alignments with guide-trees based on Jarvis and Prum. Similarity for every cell of the matrix is based on F1 score for pairs of aligned bases found to be shared or unshared between the two alignments.

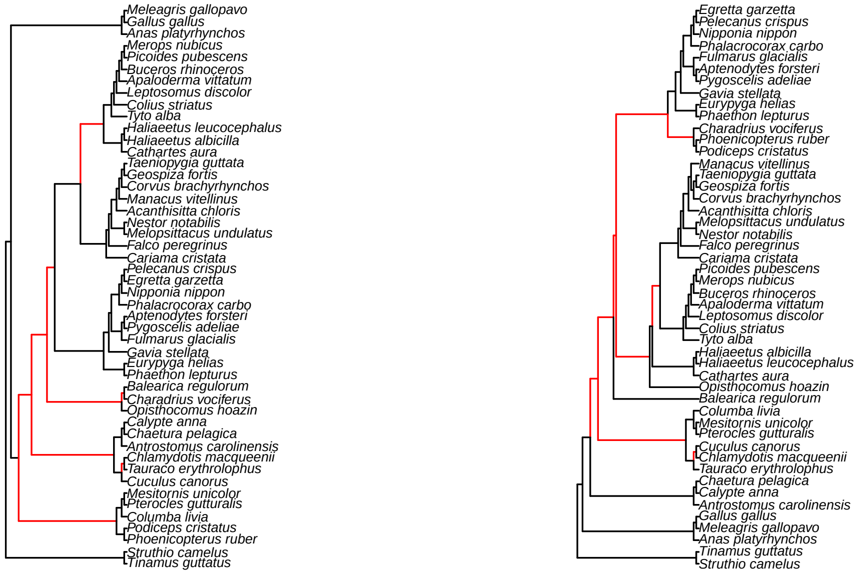

Supplementary Figure 3: Comparison between Jarvis (left) and Prum (right) topologies (branch lengths not to scale), with branches above clades not shared between the two topologies highlighted in red.

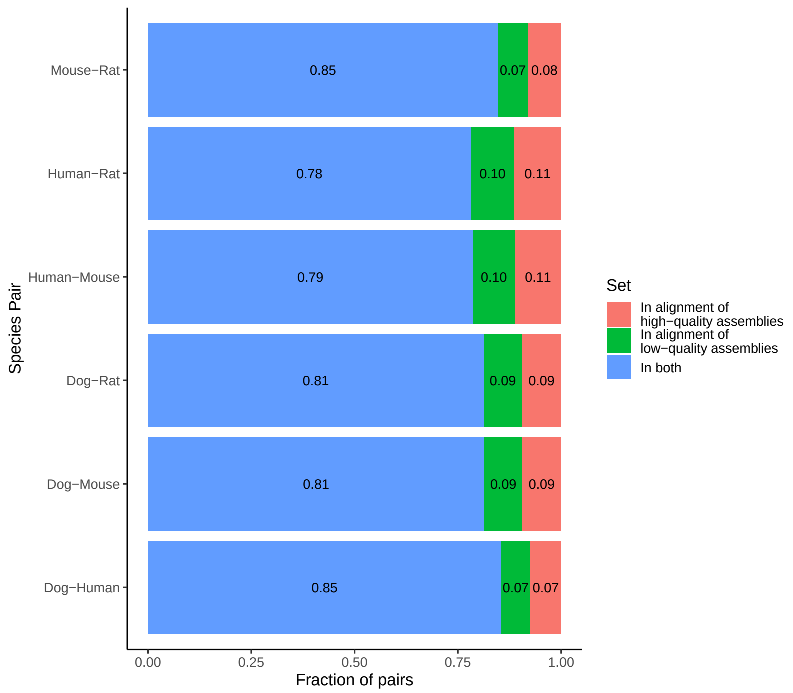

Supplementary Figure 4: Fraction of aligned pairs found only in the alignment of high-quality assemblies, only in the alignment of low-quality assemblies, or in both. Only human, mouse, rat, and dog pairs are shown since these are the only species represented by the same assemblies in both alignments.

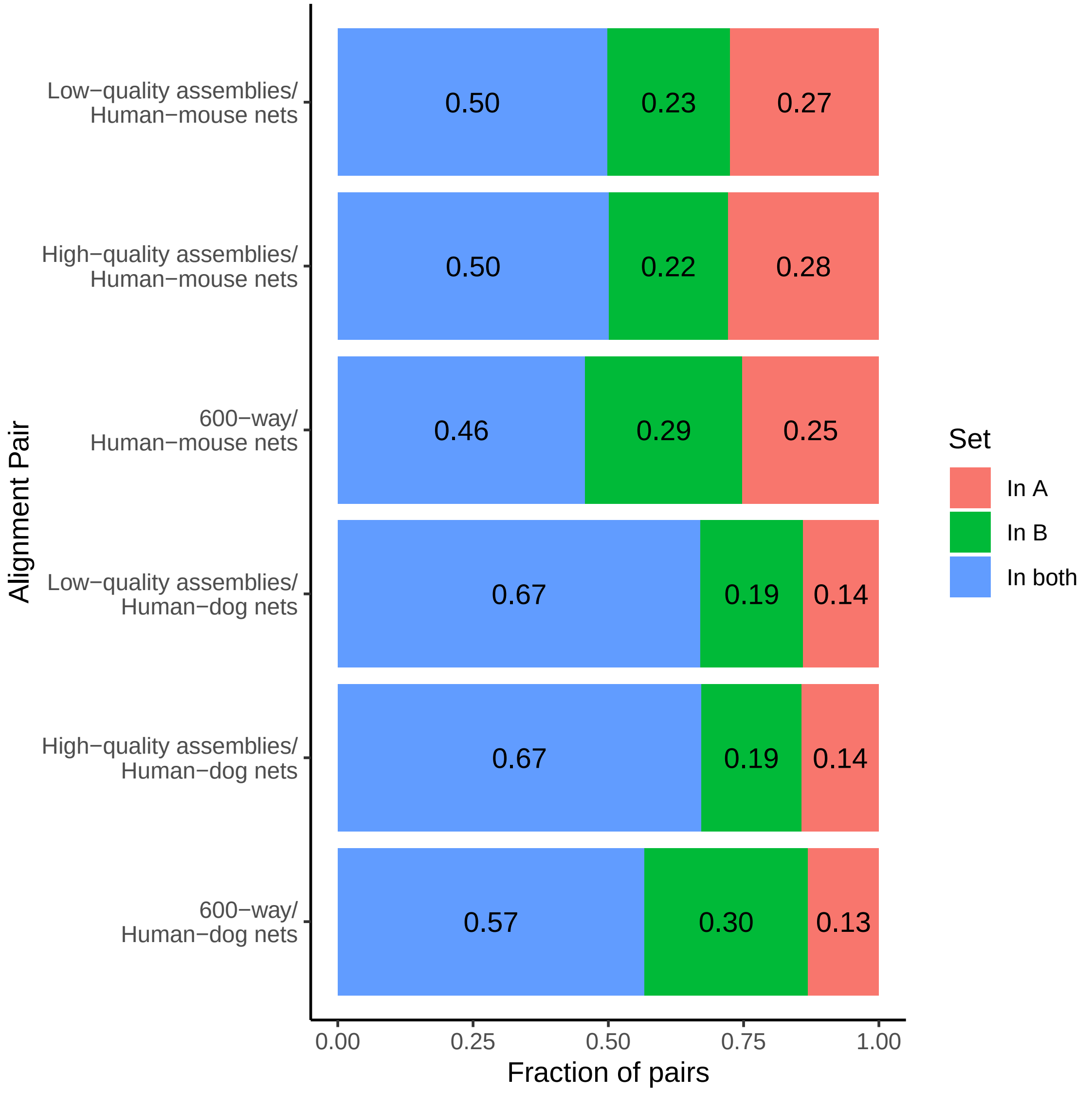

Supplementary Figure 5: Comparison of aligned pairs between human-dog and human-mouse aligned pairs within the alignments of high- and low-quality assemblies, as well as the 600-way, to those using the respective chains and nets. The Cactus alignments are filtered using the mafDuplicateFilter tool, which chooses the single closest matching sequence from each species in each alignment block to the consensus sequence of the block. This allows a fair comparison against chains and nets, which are single-copy (and therefore have no duplicates). The first alignment mentioned is referred to as A, the second is referred to as B.

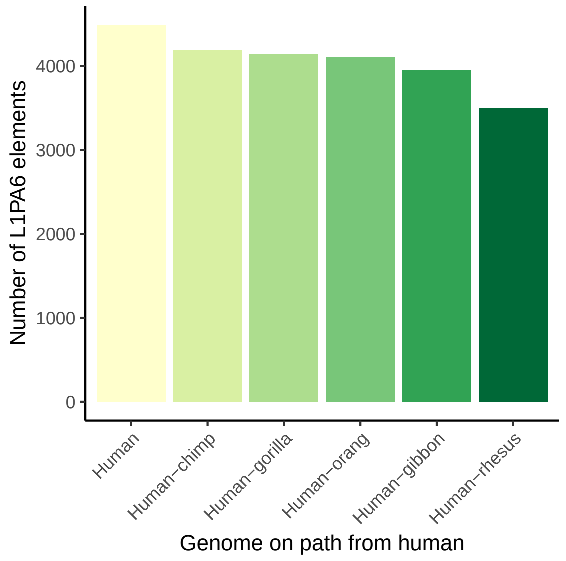

Supplementary Figure 6: Number of L1PA6 elements within ancestral genomes.

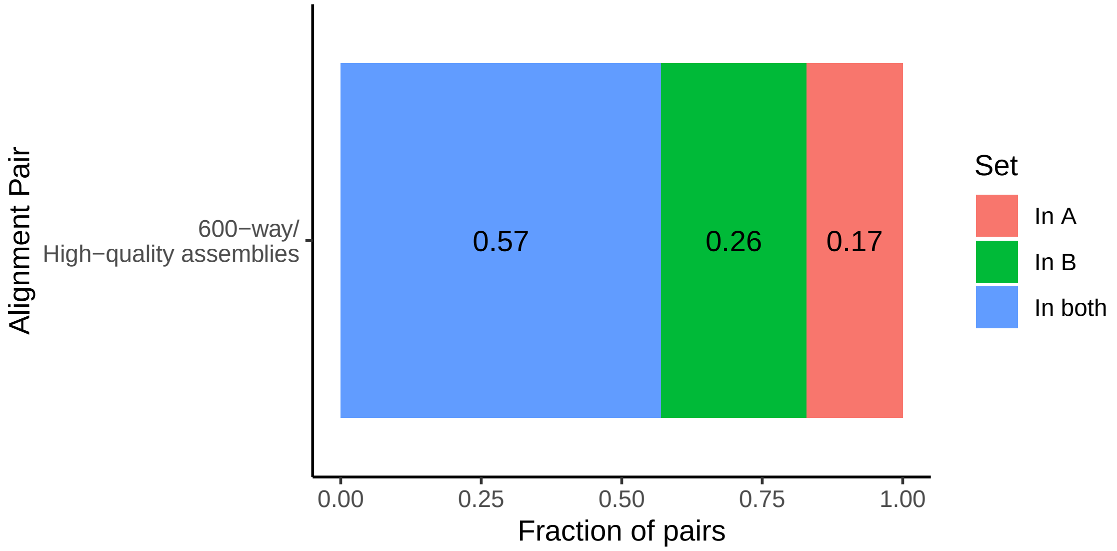

Supplementary Figure 7: Comparison of aligned pairs between the induced human/mouse/rat/dog subsets of the high-quality assemblies alignment and the 600-way. The first alignment mentioned is referred to as A, the second is referred to as B.

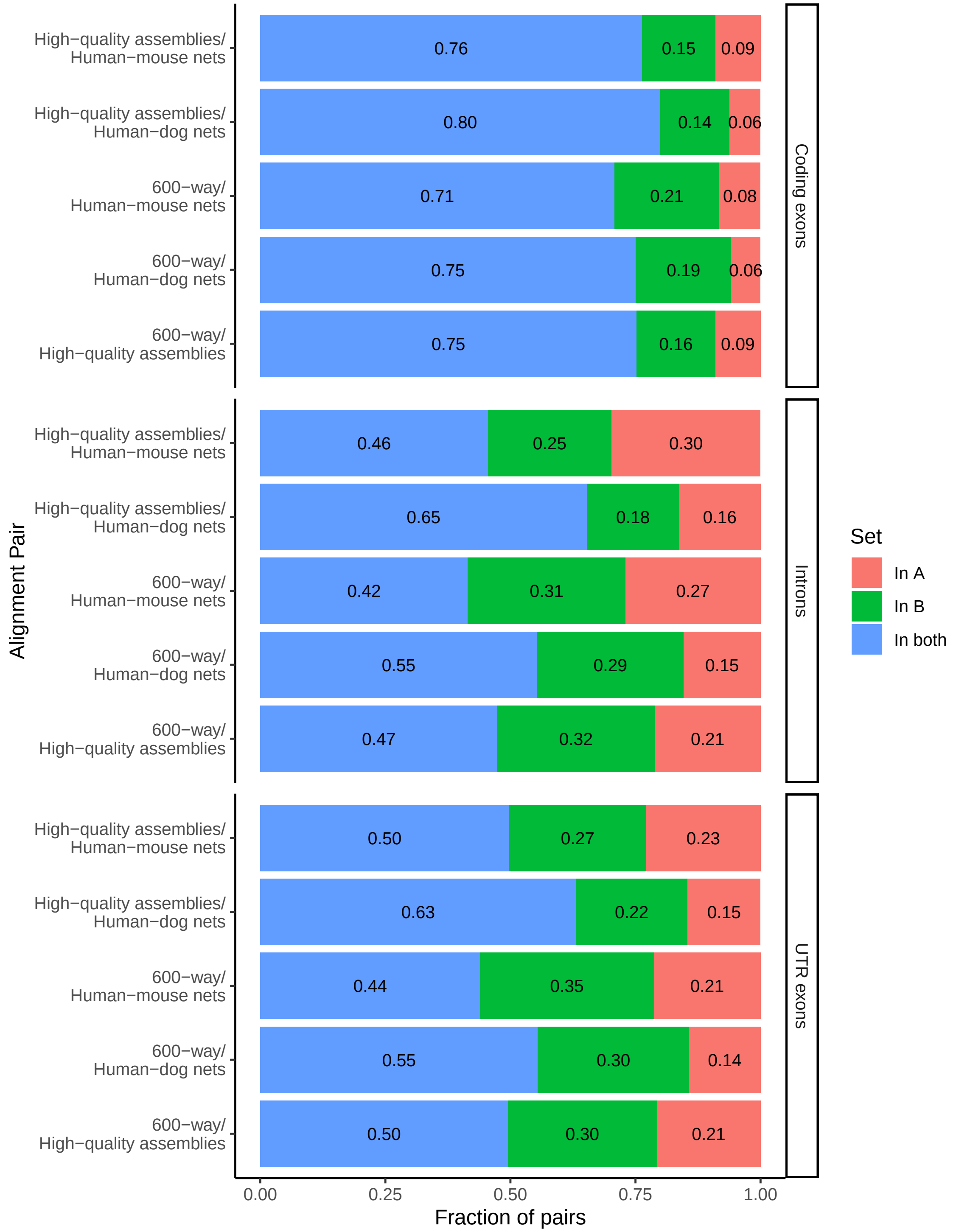

Supplementary Figure 8: Aligned pairs shared within specific regions (defined on the human reference) between several pairs of alignments. For Cactus alignments, duplicates have been removed for better comparison against the single-copy net alignments.

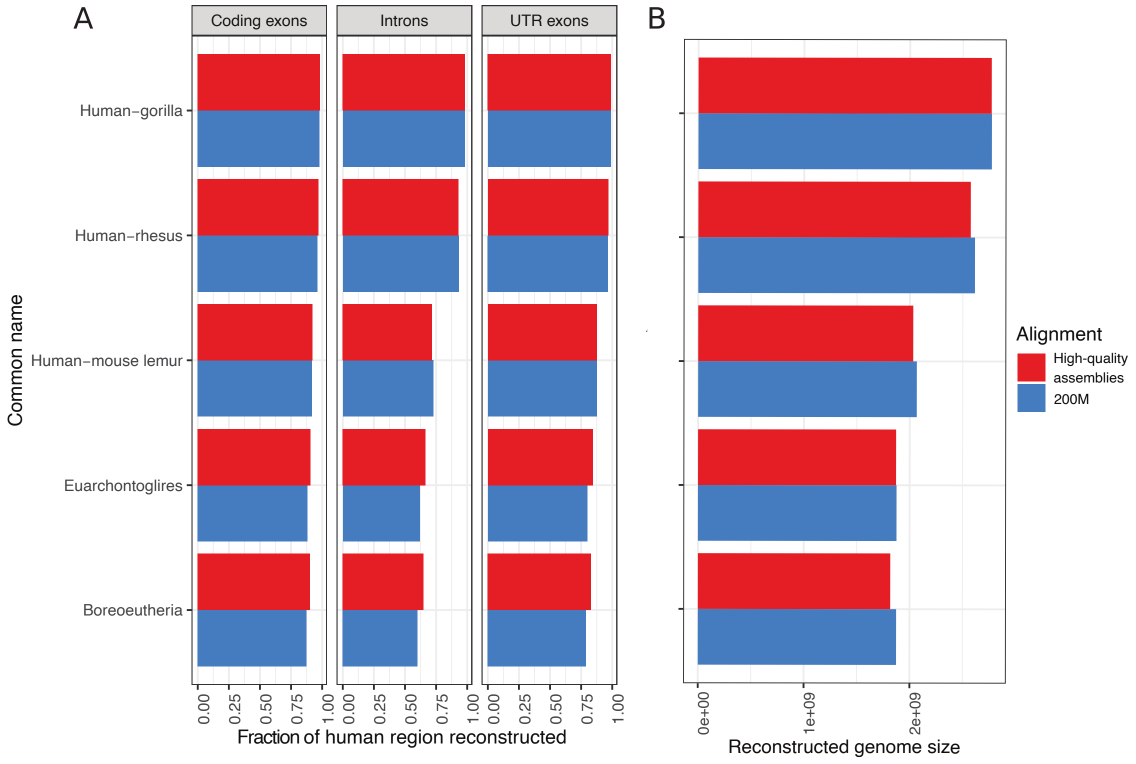

Supplementary Figure 9: Comparison of ancestors at the same position in the tree in a large (242-species Zoonomia alignment, labeled as "200M") and small (11-species, labeled as "High-quality assemblies") alignment of mammalian genomes. The smaller alignment used for comparison is the alignment of high-quality assemblies mentioned in earlier sections. A: The fraction of various types of human regions mappable to each ancestor within each alignment. B: The total size of each ancestral assembly within each alignment.

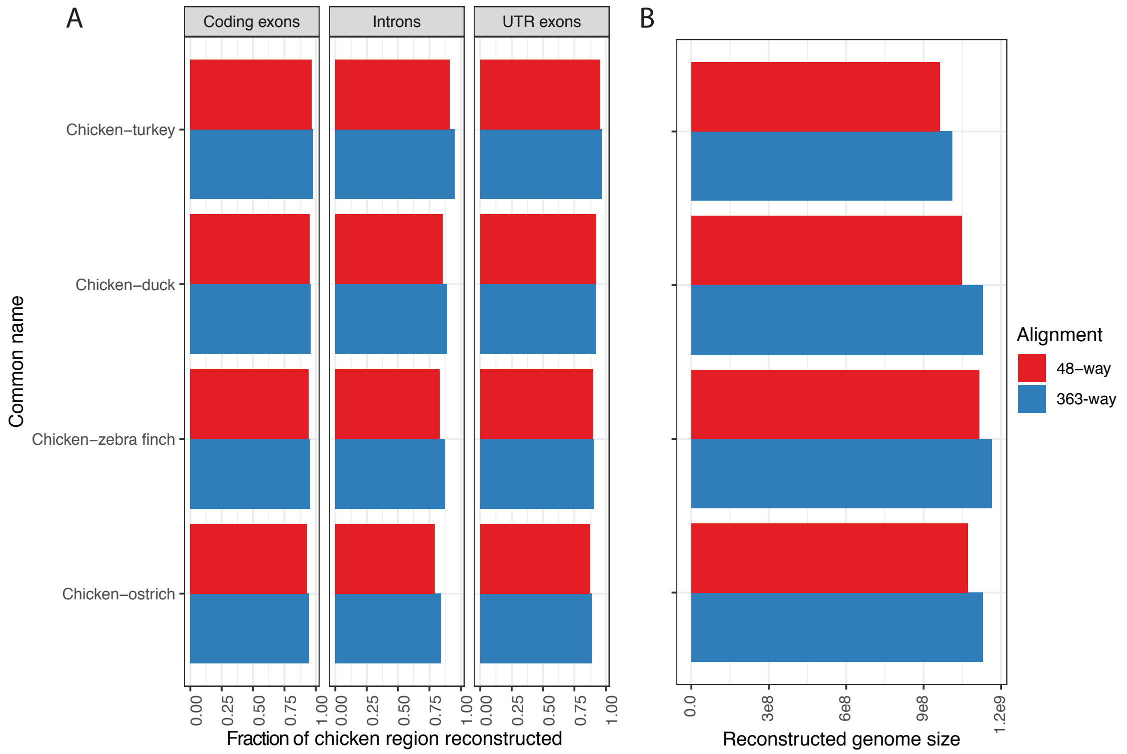

Supplementary Figure 10: Comparison of ancestors at the same position in the tree in a large (363-species, labeled as "363-way") and smaller (48-species, labeled as "48-way") alignment of bird genomes. A: The fraction of various types of chicken regions mappable to each ancestor within each alignment. B: The total size of each ancestral assembly within each alignment.

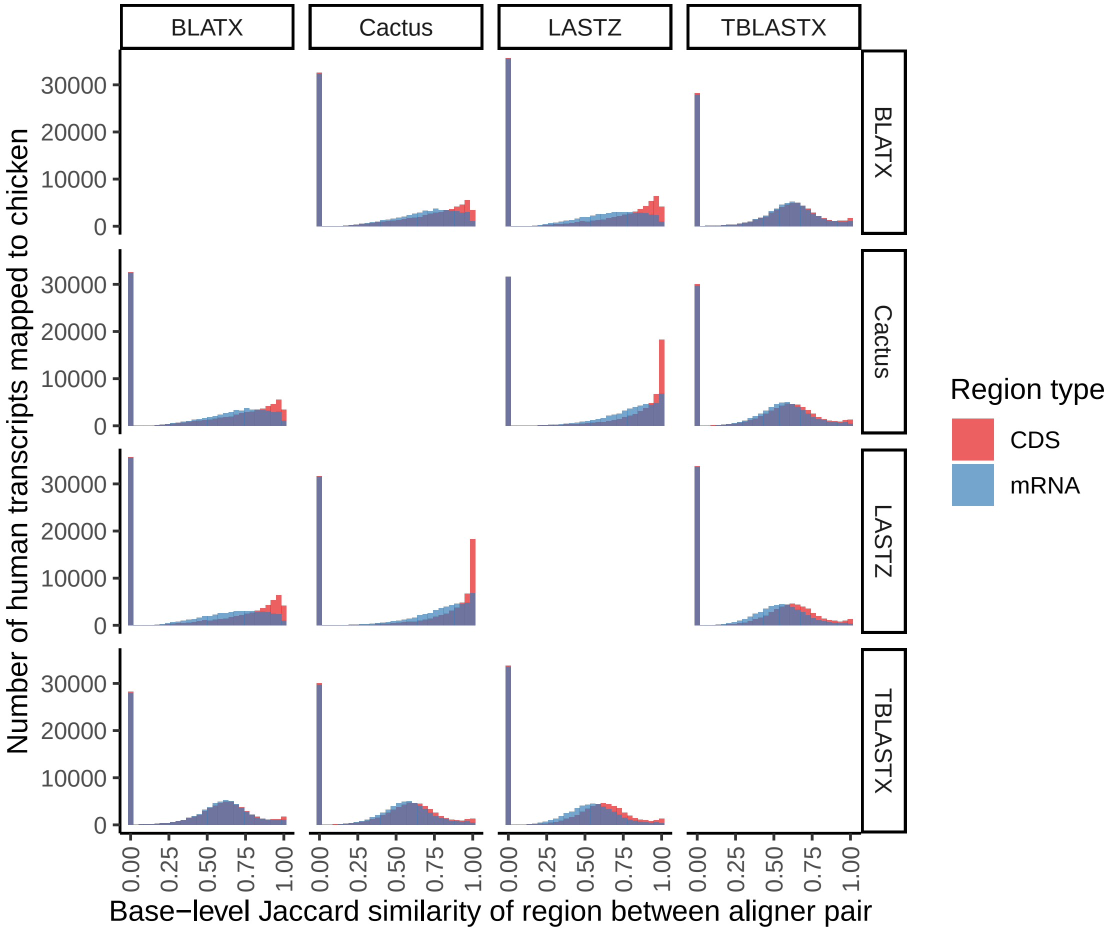

Supplementary Figure 11: Distribution of Jaccard similarities between each pair of aligners for the mRNA and coding regions of each human transcript mapped to the chicken genome (see Methods). Where one or both aligners produces multiple mappings per transcript we pick the pair of mappings (one from each mapper) with highest overlap. If one or both methods method didn't produce an alignment, a Jaccard index of 0.0 is assigned.

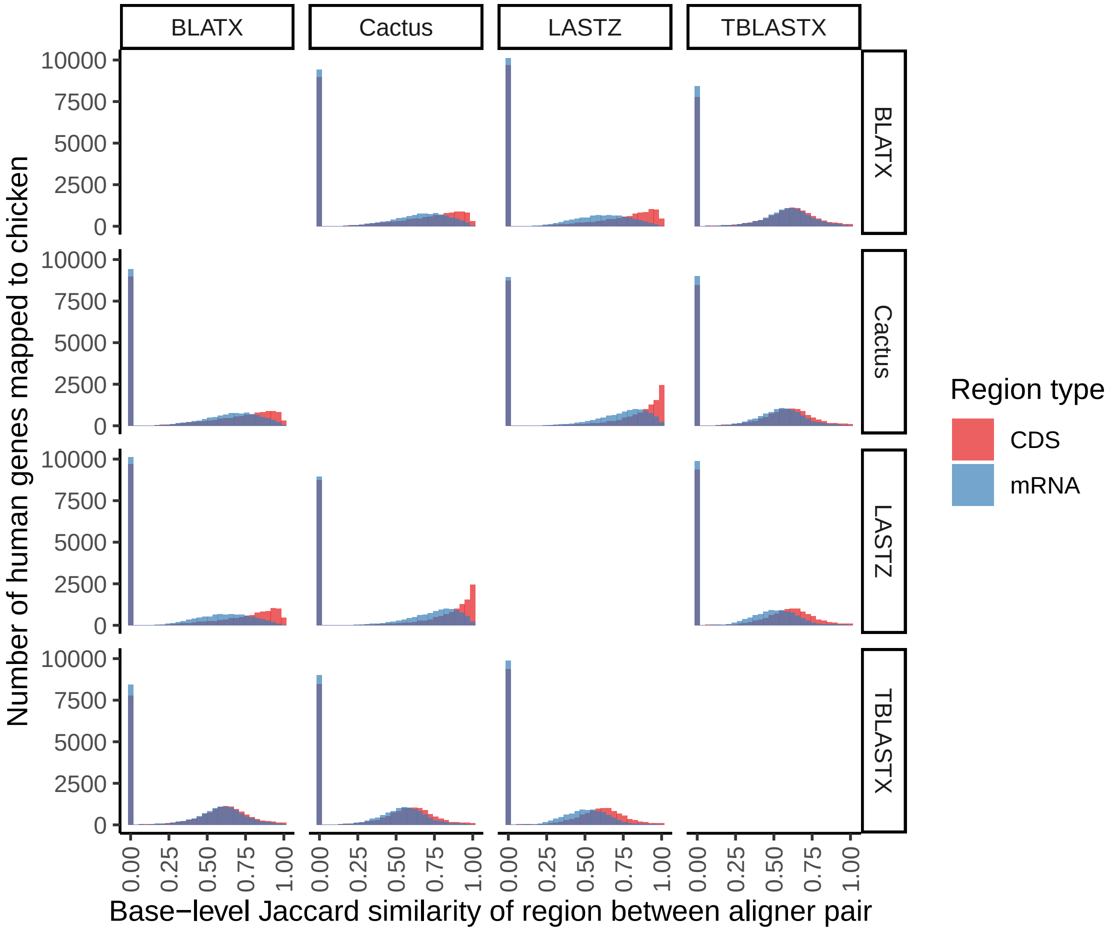

Supplementary Figure 12: Distribution of Jaccard similarities between each pair of aligners for the mRNA and coding regions of each human of each human gene mapped to the chicken genome (see Methods). Where one or both aligners produces multiple mappings per gene we pick the pair of mappings (one from each mapper) with highest overlap. If one or both methods didn't produce an alignment, a Jaccard index of 0.0 is assigned. Here gene coordinates are defined by the longest single mRNA or CDS per gene.

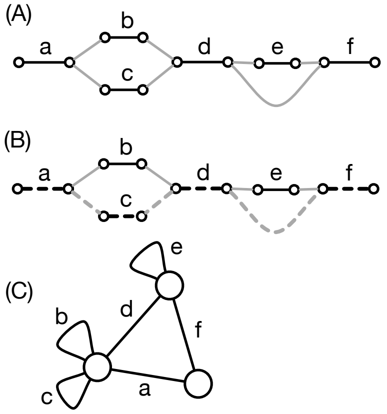

Supplementary Figure 13: Sequence and cactus graph example. (A) A biedged sequence graph constructed from the strings ‘a b d e f’, ‘a c d e f’ and ‘a b d f’, where here homology is indicated by common alphabet characters. In Progressive Cactus the sequence edges (black lines) represent alignment blocks. The adjacency edges (grey lines) indicate the sequence relationships. (B) Each input string is encoded as a restricted form of walk in the sequence graph; the path for the string ‘a c d f’ is highlighted by dotted edges. (c) The cactus graph constructed from the sequence graph using the Cactus construction procedure (see [1] for details). The subsequence ‘a d f’ common to all the input strings is represented by a simple cycle, termed a chain. The remaining substrings ‘b’, ‘c’, and ‘e’ are each in trivial chains represented using self loops.

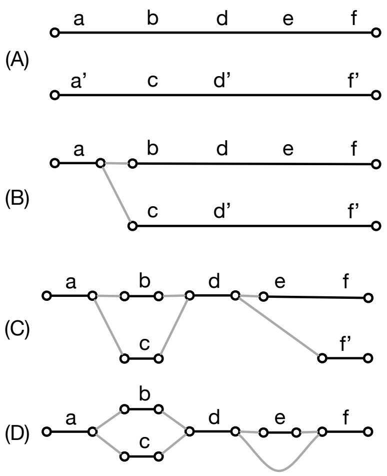

Supplementary Figure 14: Iteratively building a sequence graph by progressive glueing operations. (A) Two sequence edges labeled with their respective strings. (B) A local alignment glues together the ‘a’ copies. (C) The glueing of ‘d’. (D) The glueing of ‘f’ creates the final set of alignment blocks.

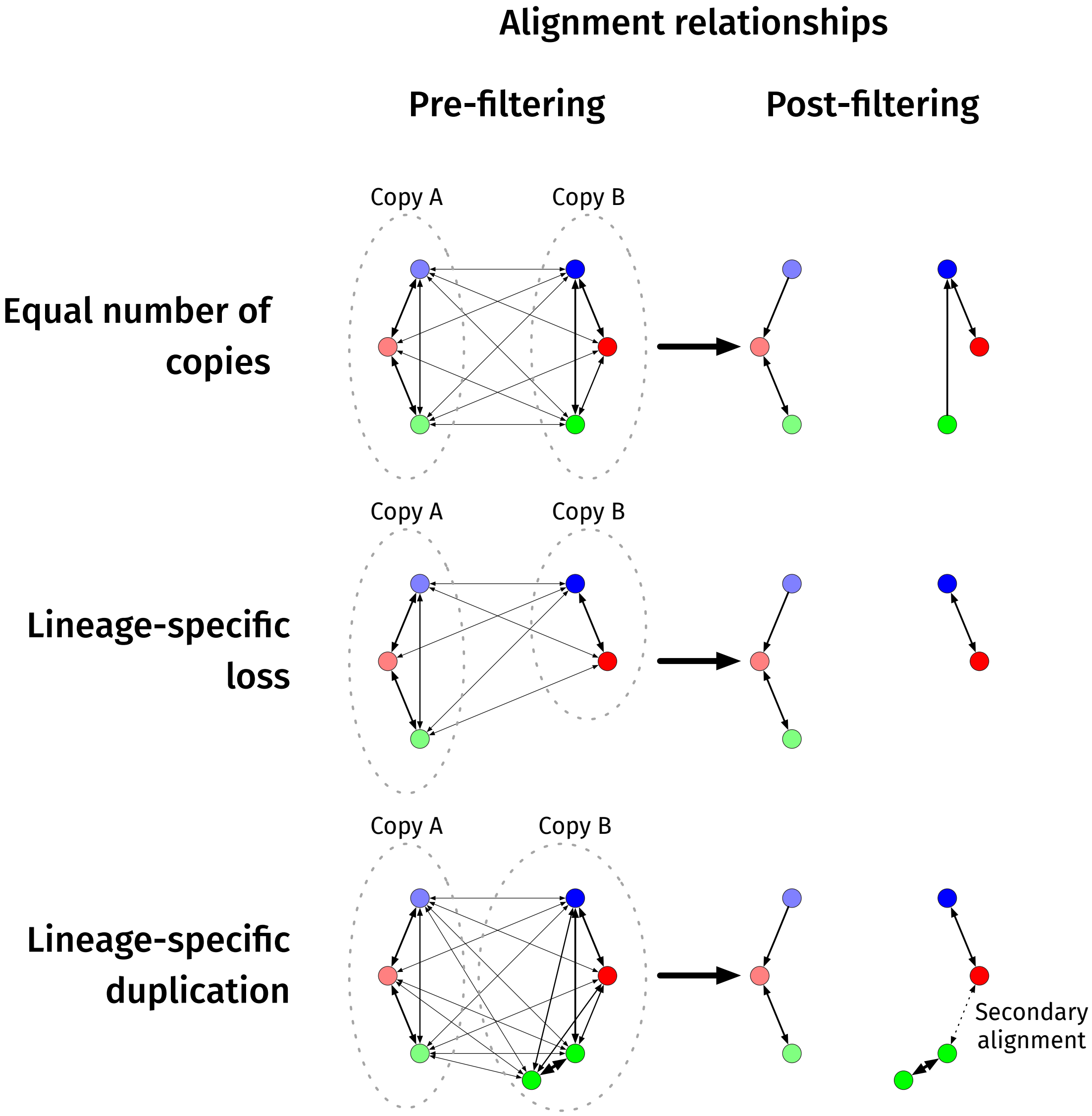

Supplementary Figure 15: A visualization of the best-hit filtering method. Here, each node of the directed graph indicates a single base, and edges represent pairwise alignment relationships (the color of the node indicates the species the base belongs to, and higher thickness of edges represents higher scores of the pairwise alignments). Since Progressive Cactus's alignment columns represent the transitive closure of the input pairwise alignment relationships, the final alignment relationships will be represented by connected components within this graph. Taking the single best hit (so that this graph contains at most one outgoing edge per base) results in the correct separation between copies if orthologous copies have higher score, but some lineage-specific duplications require secondary, non-best-hit alignments to bring together orthologs from different species.

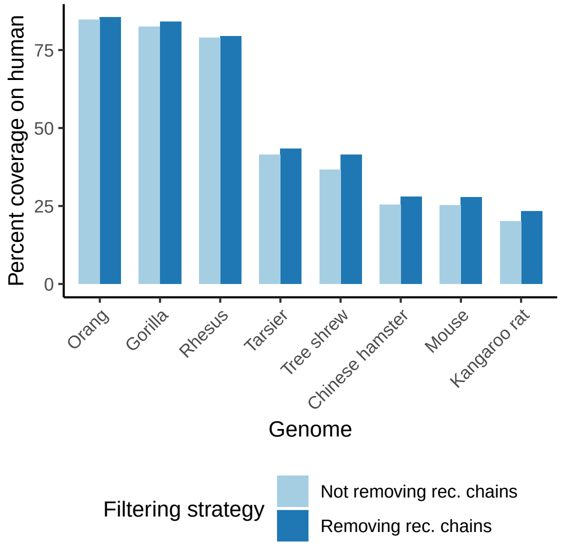

Supplementary Figure 16: Coverage of the human genome from alignments with and without removing recoverable chains after the CAF process. While the coverage is increased overall across all genomes when removing recoverable chains, the increase is relatively larger in more distant species.

Aligner	Alignathon entry name	Precision	Recall	F1
Progressive Cactus (this manuscript)	—	0.730	0.873	0.795
Cactus (Alignathon version)	`cactus`	0.706	0.885	0.785
VISTA-LAGAN [2]	`brudno`	0.619	0.791	0.694
EPO [3]	`ebi.epo`	0.224	0.893	0.359
Mercator/Pecan [4, 5]	`ebi.mp`	0.368	0.878	0.519
PSAR-Align [6]	`kimMa`	0.614	0.826	0.703
AutoMZ [7]	`minmei.automz`	0.606	0.694	0.647
TBA [7]	`minmei.tba`	0.640	0.769	0.699
Mugsy [8]	`mugsy`	0.065	0.931	0.122
Robusta [9]	`robusta`	0.357	0.744	0.482
GenomeMatch	`softberry.v1`	0.104	0.980	0.188
GenomeMatch	`softberry.v2`	0.104	0.974	0.187
GenomeMatch	`softberry.v3`	0.105	0.968	0.189
MULTIZ [7]	`ucsc`	0.616	0.818	0.703

Supplementary Table 1: Precision, recall, and F1 scores for the simulated mammals dataset from the Alignathon [10]. All alignments except for Progressive Cactus are as submitted for the Alignathon.

Aligner	Alignathon entry name	Precision	Recall	F1
Progressive Cactus (this manuscript)	—	0.986	0.991	0.989
Cactus (Alignathon version)	`cactus`	0.984	0.983	0.983
VISTA-LAGAN [2]	`brudno`	0.978	0.983	0.980
Mercator/Pecan [4, 5]	`compara`	0.940	0.996	0.967
PSAR-Align [6]	`kimMa`	0.980	0.995	0.988
AutoMZ [7]	`minmei.automz`	0.980	0.992	0.986
TBA [7]	`minmei.tba`	0.981	0.992	0.986
Mugsy [8]	`mugsy`	0.978	0.996	0.987
progressiveMauve [11]	`pmauve`	0.971	0.997	0.984
Robusta [9]	`robusta`	0.941	0.986	0.963
GenomeMatch	`softberry.v1`	0.898	0.997	0.945
GenomeMatch	`softberry.v2`	0.898	0.972	0.934
GenomeMatch	`softberry.v3`	0.905	0.261	0.405
MULTIZ [7]	`ucsc`	0.980	0.992	0.986

Supplementary Table 2: Precision, recall, and F1 scores for the simulated primates dataset from the Alignathon [10].

Alignment	URL
Jarvis	`https://s3.amazonaws.com/alignment-output/cactus48BIRDS_jarvis14.hal`
Prum	`https://s3.amazonaws.com/alignment-output/cactus48BIRDS_prum15.hal`
Consensus	`https://s3.amazonaws.com/alignment-output/cactus48BIRDS_consensus.hal`
Permuted	`https://s3.amazonaws.com/alignment-output/cactus48BIRDS_permute.hal`

Supplementary Table 3: Alignments used in the guide-tree analysis.

Species	Low-quality assembly alignment			High-quality assembly alignment		
	Assembly	Scaffold N50	Contig N50	Assembly	Scaffold N50	Contig N50
Gorilla	gorGor3	913,958	11,691	Susie3	20,634,945	9,406,846
Mouse lemur	micMur1	140,884	3,511	Mmur_3.0	108,171,978	210,702
Chinese hamster	criGri1	1,558,295	27,129	criGriCHOV2	62,039,716	97,133
Pig	susScr3	576,008	69,503	susScr11	88,231,837	48,231,277
Horse	equCab2	46,749,900	112,381	equCab3	87,230,776	1,502,753
Rhesus	rheMac8	4,193,270	107,172	rheMac10	82,346,004	46,608,966
Camel	ASM164081v1	31,503	31,503	CamDro3	70,369,702	236,391

Supplementary Table 4: Assembly versions used in the alignments of low-quality and high-quality assemblies. In addition to these 7 genomes, 4 others were included in each alignment with the same assemblies in both: human (GRCh38), mouse (mm10), rat (rn6), and dog (canFam3).

Genome	Coverage on the human genome	
	Alignment of high-quality assemblies	Alignment of low-quality assemblies
Human	1.00	1.00
Gorilla	0.90	0.85
Rhesus	0.83	0.82
Mouse lemur	0.54	0.43
Horse	0.51	0.50
Dog	0.47	0.47
Pig	0.46	0.42
Camel	0.46	0.46
Chinese hamster	0.34	0.33
Mouse	0.33	0.32
Rat	0.33	0.32

Supplementary Table 5: Coverage on the human genome in the high-quality vs. low-quality assembly alignments.

	Human to chicken			
Category	*BLATX*	*TBLASTX*	*LASTZ*	*Cactus*
Source transcripts	84,001	84,001	84,001	84,001
Mapped	58,574	67,197	56,335	61,925
Rate mapped	0.70	0.80	0.67	0.74
Multi-mapped	9,605	34,573	1,037	1,634
Rate multi-mapped	0.16	0.51	0.02	0.03
Mean base mapping rate	0.33	0.33	0.44	0.42
Median base mapping rate	0.32	0.33	0.48	0.43
Mean CDS base mapping rate	0.50	0.48	0.60	0.60
Median CDS base mapping rate	0.60	0.56	0.85	0.79

Supplementary Table 6: Comparison of mapping protein-coding transcripts from human to chicken using four alignment and mapping methods (see Methods). Human source transcripts are from GENCODE V34. The multi-mapped rate is computed for those source transcripts that have any mappings. The base mapping rates are computed for the single best mapping of each mapped transcript. The best mapping is defined as the mapping with the highest average of the number of bases mapped across (i) the entire transcript and (ii) just the CDS portion.

Method	source mRNAs	mRNAs aligned	align count	mean ident	median ident	mean aligned	median aligned
BLATX	84,001	58,574	71,691	0.77	0.76	0.45	0.42
TBLASTX	84,001	67,197	137,270	0.66	0.65	0.34	0.31
LASTZ	84,001	56,335	57,496	0.74	0.74	0.66	0.68
Cactus	84,001	61,925	63,607	0.75	0.76	0.56	0.56

Supplementary Table 7: Transcript alignment statistics mapping from human to chicken (see Methods). For each alignment method, this shows the number of mRNAs that were aligned (mRNAs aligned) and the total number of alignments of those mRNA (align count), along with the mean and median of the nucleotide identity and of the fraction of the mRNAs nucleotides that aligned.

Method	source mRNAs	mRNAs aligned	align count	mean ident	median ident	mean aligned	median aligned
BLATX	19,695	12,102	15,619	0.74	0.75	0.36	0.32
TBLASTX	19,695	13,968	29,011	0.63	0.62	0.30	0.27
LASTZ	19,695	11,935	12,317	0.72	0.72	0.56	0.55
Cactus	19,695	12,971	13,227	0.74	0.74	0.46	0.43

Supplementary Table 8: Gene alignment statistics mapping from human to chicken (see Methods). Here gene coordinates are defined by the longest single transcript per gene. For each method, this shows the number of mRNAs that were aligned (mRNAs aligned) and the total number of alignments of those mRNA (align count), along with the mean and median of the nucleotide identity and of the fraction of the mRNAs nucleotides that aligned.

Category	Gene Counts				CDS Base Counts			
	LASTZ		*Cactus*		*LASTZ*		*Cactus*	
	count	rate	count	rate	count	rate	count	rate
source	19,695	1.00	19,695	1.00	34,356,456	1.00	34,356,456	1.00
missing	3,485	0.18	3,429	0.17	4,141,477	0.12	3,967,426	0.12
unmapped	4,110	0.21	2,991	0.15	6,052,342	0.18	4,083,397	0.12
mapped hit	10,832	0.55	11,758	0.60	15,696,629	0.46	15,732,865	0.46
mapped miss	194	0.01	387	0.02	7,499,854	0.22	9,661,280	0.28
mapped only	1,074	0.05	1,130	0.06	966,154	0.03	911,488	0.03

Supplementary Table 9: Comparison of each of the *LASTZ* and *Cactus* alignments to the union of the *BLATX* and *TBLASTX* translated alignments. This analysis implicitly uses the union of the translated alignments as a proxy to a truth set to compare the non-translated methods. Human coding sequences from GENCODE V34 are used, picking the coding sequence from the longest transcript per gene to define a minimally overlapping set. To account for human bases which map to multiple bases in chicken (which occurs frequently for the translated alignment methods that include very distant, fragmented, paralogous alignments, but much less often for the non-translated methods), when per CDS there is either or both multiple translated alignments or multiple non-translated alignments, we pick the pair of mappings (one translated, one from the non-translated method) with highest pairwise Jaccard similarity. Base level counts are then reported for this pairing of the CDS. We report numbers in terms of genes and individual human coding bases. The *source* row is the total number of human genes/coding bases. The *missing* counts are the number of human genes/coding bases that were not mapped by either the untranslated method (*Cactus* or *LASTZ*) or the one of the translated alignment methods. The *unmapped* are cases where there are translated alignments and no untranslated alignment. The *mapped hit* are cases where the untranslated alignment is the same as a translated alignment. With *mapped miss*, the untranslated alignments do not overlap any of the translated alignments. The *mapped only* are counts of genes/bases that are only aligned by the untranslated aligner.

Methods		Transcripts to chicken		Genes to chicken	
		mRNA	CDS	mRNA	CDS
BLATX	*TBLASTX*	0.41	0.42	0.34	0.37
BLATX	*LASTZ*	0.39	0.45	0.30	0.38
BLATX	*Cactus*	0.43	0.46	0.34	0.40
TBLASTX	*LASTZ*	0.33	0.37	0.26	0.32
TBLASTX	*Cactus*	0.37	0.39	0.30	0.34
LASTZ	*Cactus*	0.50	0.54	0.41	0.47

Supplementary Table 10: Similarity of mapping protein-coding transcripts and genes between human and chicken using the four alignment and mapping methods (see Methods). The metric is the mean Jaccard index computed at the base-level of individual transcripts. Where one or both aligners produces multiple mappings per transcript we pick the pair of mappings (one from each mapper) with highest overlap. If one or both methods method didn't produce an alignment, a Jaccard index of 0.0 is assigned.

	Transcripts		Genes	
Method	mRNA	CDS	mRNA	CDS
BLATX	0.34	0.47	0.57	0.42
TBLASTX	0.24	0.32	0.41	0.31
LASTZ	0.43	0.58	0.63	0.56
Cactus	0.38	0.53	0.64	0.51

Supplementary Table 11: The Jaccard index similarity metric of human protein-coding transcript and gene mappings to the native chicken transcript annotations for the four alignment methods For each source that was mapped, we pick the target native annotation with the highest base-level Jaccard index to any of the source transcript's mappings as the candidate ortholog. This table reports the mean Jaccard index for each mapped source transcript and the chosen target transcript. The preliminary state of the chicken annotation as compared to human limits the value of making absolute interpretations of the mappings' correctness. In particular, this analysis doesn't account for alignment to paralogous genes. However, it provides a useful, if limited, relative comparison. Due to this asymmetry in the sets (chicken has just 13,391 transcripts) and not using independent ortholog assignment, we only consider those alignments that actually overlap, not the non-overlapping or non-mapping annotations. This type of in depth ortholog comparison would be of great value, however it is beyond the scope of this paper.

Alignment	URL
With improved filtering	`https://alignment-output.s3.amazonaws.com/10plusway-mapq.hal`
Without improved filtering	`https://alignment-output.s3.amazonaws.com/10plusway-master.hal`

Supplementary Table 12: Alignments compared in the paralogy-filtering evaluation.

Genome	UCSC assembly version
Tree shrew	tupChi1
Kangaroo rat	dipOrd1
Human	hg38
Chimp	panTro6
Rhesus	rheMac8
Mouse	mm10
Rat	rn6
Dog	canFam3
Cat	felCat8
Pig	susScr11
Cow	bosTau8
Horse	equCab3
Elephant	loxAfr3

Supplementary Table 13: Assemblies used in the paralogy-filtering evaluation.

Genome	Coding genes missing from final set		Coding transcripts missing from final set	
	Outgroup filtering	Best-hit filtering	Outgroup filtering	Best-hit filtering
Chimpanzee	1716	1612	6244	5872
Gorilla	1829	1647	6469	6100

Supplementary Table 14: Number of human genes / transcripts that have no assigned ortholog in the "consensus" CAT gene set across the different alignments.

Genome	Transcript projections filtered during initial pass	
	Chimpanzee	Gorilla
Outgroup filtering	43709	31678
Best-hit filtering	13567	15765

Supplementary Table 15: Number of transcripts filtered out in the initial `pslCDnaFilter` step of CAT, which attempts to remove paralogs and processed pseudogenes.

References

[1] Paten, B. *et al.* Cactus graphs for genome comparisons. *J. Comput. Biol.* **18**, 469–481 (2011).

[2] Dubchak, I., Poliakov, A., Kislyuk, A. & Brudno, M. Multiple whole-genome alignments without a reference organism. *Genome Res.* **19**, 682–689 (2009).

[3] Paten, B., Herrero, J., Beal, K., Fitzgerald, S. & Birney, E. Enredo and Pecan: genome-wide mammalian consistency-based multiple alignment with paralogs. *Genome Res.* **18**, 1814–1828 (2008).

[4] Dewey, C. N. Aligning multiple whole genomes with Mercator and MAVID. *Methods Mol. Biol.* **395**, 221–236 (2007).

[5] Paten, B., Herrero, J., Beal, K. & Birney, E. Sequence progressive alignment, a framework for practical large-scale probabilistic consistency alignment. *Bioinformatics* **25**, 295–301 (2009).

[6] Kim, J. & Ma, J. PSAR-align: improving multiple sequence alignment using probabilistic sampling. *Bioinformatics* **30**, 1010–1012 (2014).

[7] Blanchette, M. *et al.* Aligning multiple genomic sequences with the threaded blockset aligner. *Genome Research* **14**, 708–715 (2004).

[8] Angiuoli, S. V. & Salzberg, S. L. Mugsy: fast multiple alignment of closely related whole genomes. *Bioinformatics* **27**, 334–342 (2011).

[9] Notredame, C. Robusta: a meta-multiple genome alignment tool. `http://www.tcoffee.org/Projects/robusta/`.

[10] Earl, D. *et al.* Alignathon: a competitive assessment of whole-genome alignment methods. *Genome research* **24**, 2077–2089 (2014).

[11] Darling, A. E., Mau, B. & Perna, N. T. Progressivemauve: Multiple genome alignment with gene gain, loss and rearrangement. *PLoS ONE* **5** (2010).
