# Supplementary material for: Progressive Cactus is a multiple-genome aligner for the thousand-genome era
Source: Nature. 2020 Nov 11;587(7833):246–51. doi: 10.1038/s41586-020-2871-y (PMC7673649; doi:10.1038/s41586-020-2871-y)
Supplement: Supplementary file 2 — Reporting Summary [file 41586_2020_2871_MOESM2_ESM.pdf]

nature research

Corresponding author(s): Benedict Paten, Guojie Zhang

Last updated by author(s): 6/28/2020

Reporting Summary

Nature Research wishes to improve the reproducibility of the work that we publish. This form provides structure for consistency and transparency in reporting. For further information on Nature Research policies, see Authors & Referees and the Editorial Policy Checklist .

Statistics

For all statistical analyses, confirm that the following items are present in the figure legend, table legend, main text, or Methods section.

n/a	Confirmed	
☐	☒	The exact sample size (*n*) for each experimental group/condition, given as a discrete number and unit of measurement
☒	☐	A statement on whether measurements were taken from distinct samples or whether the same sample was measured repeatedly
☒	☐	The statistical test(s) used AND whether they are one- or two-sided<br>*Only common tests should be described solely by name; describe more complex techniques in the Methods section.*
☒	☐	A description of all covariates tested
☒	☐	A description of any assumptions or corrections, such as tests of normality and adjustment for multiple comparisons
☐	☒	A full description of the statistical parameters including central tendency (e.g. means) or other basic estimates (e.g. regression coefficient) AND variation (e.g. standard deviation) or associated estimates of uncertainty (e.g. confidence intervals)
☒	☐	For null hypothesis testing, the test statistic (e.g. *F*, *t*, *r*) with confidence intervals, effect sizes, degrees of freedom and *P* value noted<br>*Give P values as exact values whenever suitable.*
☒	☐	For Bayesian analysis, information on the choice of priors and Markov chain Monte Carlo settings
☒	☐	For hierarchical and complex designs, identification of the appropriate level for tests and full reporting of outcomes
☒	☐	Estimates of effect sizes (e.g. Cohen's *d*, Pearson's *r*), indicating how they were calculated

Our web collection on statistics for biologists contains articles on many of the points above.

Software and code

Policy information about availability of computer code

Data collection	No software was used to collect data
Data analysis	The Cactus software (https://github.com/comparativegenomicstoolkit/cactus) was used to analyze the assemblies and generate the alignments described. The archived version 1.0 is here: https://doi.org/10.5281/zenodo.3873410 Specifics commit used: 51eb980b, 36304707, 450da74, aca859f, 56874bde and 49e80082. These specific commits are referenced to individual analyses in the Methods.<br><br>In addition we used software from the following URLs (all referenced in the methods):<br>https://github.com/dentearl/evolverSimControl, commit b3236deb<br>https://github.com/dentearl/mafTools, commit 82077ac3<br>https://github.com/dentearl/mwgAlignAnalysis, commit df98753<br>https://github.com/joelarmstrong/repeatMaskerPipeline, commit a6ad966<br>https://github.com/rmhubley/RepeatMasker, commit 2d947604<br>http://evolution.genetics.washington.edu/phylip/getme-new1.html , version 3.695<br>https://github.com/ComparativeGenomicsToolkit/Comparative-Annotation-Toolkit, commit 7a8c7e24<br>https://github.com/ComparativeGenomicsToolkit/toil, commit 7a8c7e24<br>https://github.com/CshlSiepelLab/phast, commit 52e8de9<br>https://github.com/marbl/Mash, commit 541971b<br>https://github.com/ucscGenomeBrowser/kent, commit 8a8d921<br>https://github.com/ComparativeGenomicsToolkit/hal, commit 68db41d<br>ftp://ftp.ncbi.nlm.nih.gov/blast/executables/blast+/2.10.0/, version tblastn: 2.10.0<br>http://www.microbesonline.org/fasttree/, version 2.1.11<br>https://github.com/lastz/lastz, version 1.03.54

For manuscripts utilizing custom algorithms or software that are central to the research but not yet described in published literature, software must be made available to editors/reviewers. We strongly encourage code deposition in a community repository (e.g. GitHub). See the Nature Research guidelines for submitting code & software for further information.

Data

Policy information about availability of data

All manuscripts must include a data availability statement. This statement should provide the following information, where applicable:

- Accession codes, unique identifiers, or web links for publicly available datasets
- A list of figures that have associated raw data
- A description of any restrictions on data availability

The 600-way genome alignment is comprised of data gathered for the Zoonomia project and
data from the Bird 10,000 genomes (B10K) project. All genomes have been archived in GenBank, spreadsheets containing all the accession numbers of the assemblies is provided in the supplementary material.

All the alignment data is made available for immediate public use. The 600-way alignment is available in HAL format at https://alignmentoutput.s3.amazonaws.com/600way.hal. We also provide the subset of the alignment containing the Zoonomia genomes at: https://alignment-output.s3.amazonaws.com/200m-v1.hal. The subset of the alignment containing the Bird 10K genomes is at: https://alignment-output.s3.amazonaws.com/birds-final.hal. A visualization of the alignments and associated data is available by loading our assembly hub into the UCSC browser. By copying the hub link https://comparative-genomics-hubs.s3-us-west-2.amazonaws.com/600way_hub.txt into the "Track Hubs" page, the 605 genomes and associated tracks will be available.

The guide-tree topology was taken from the TimeTree database using the release current in October 2018.

Field-specific reporting

Please select the one below that is the best fit for your research. If you are not sure, read the appropriate sections before making your selection.

[x] Life sciences [ ] Behavioural & social sciences [ ] Ecological, evolutionary & environmental sciences

For a reference copy of the document with all sections, see nature.com/documents/nr-reporting-summary-flat.pdf

Life sciences study design

All studies must disclose on these points even when the disclosure is negative.

Sample size	No sample size calculation was performed for any of the analyses.
Data exclusions	Before constructing the 600-way we evaluated the quality of publicly available genome assemblies; those that did not meet our contiguity or quality requirements were excluded and did not make it into our set of 605 genomes. Other than that, no data was excluded.
Replication	We have replicated the small simulated alignment sets we describe, which show near-identical results. For cost reasons, we have not replicated the larger alignments we describe.
Randomization	We do not believe randomization is relevant since we are not describing an analysis divided into experimental groups.
Blinding	We do not believe blinding is relevant since we are not describing an analysis divided into experimental groups.

Reporting for specific materials, systems and methods

We require information from authors about some types of materials, experimental systems and methods used in many studies. Here, indicate whether each material, system or method listed is relevant to your study. If you are not sure if a list item applies to your research, read the appropriate section before selecting a response.

Materials & experimental systems

n/a	Involved in the study	
[x]	[ ]	Antibodies
[x]	[ ]	Eukaryotic cell lines
[x]	[ ]	Palaeontology
[x]	[ ]	Animals and other organisms
[x]	[ ]	Human research participants
[x]	[ ]	Clinical data

Methods

n/a	Involved in the study	
[x]	[ ]	ChIP-seq
[x]	[ ]	Flow cytometry
[x]	[ ]	MRI-based neuroimaging
